# Grammatical Perspective-Taking in Comprehension and Production

**DOI:** 10.1162/opmi_a_00071

**Published:** 2023-02-01

**Authors:** Carolyn Jane Anderson, Brian Dillon

**Affiliations:** Department of Computer Science, Wellesley College, Wellesley, USA; Department of Linguistics, University of Massachusetts Amherst, Amherst, USA

**Keywords:** perspective, cognitive modeling, Rational Speech Acts, motion verbs

## Abstract

Language use in conversation requires conversation partners to consider each other’s points-of-view, or perspectives. A large body of work has explored how conversation partners take into account differences in knowledge states when choosing referring expressions. This paper explores how well findings from perspective-taking in reference generalize to a relatively understudied domain of perspective: the processing of grammatical perspectival expressions like the motion verbs *come* and *go* in English. We re-visit findings from perspective-taking in reference that conversation participants are subject to egocentric biases: they are biased towards their own perspectives. Drawing on theoretical proposals for grammatical perspective-taking and prior experimental studies of perspective-taking in reference, we compare two models of grammatical perspective-taking: a serial anchoring-and-adjustment model, and a simultaneous integration model. We test their differing predictions in a series of comprehension and production experiments using the perspectival motion verbs *come* and *go* as a case study. While our comprehension studies suggest that listeners reason simultaneously over multiple perspectives, as in the simultaneous integration model, our production findings are more mixed: we find support for only one of the simultaneous integration model’s two key predictions. More generally, our findings suggest a role for egocentric bias in production for grammatical perspective-taking as well as when choosing referring expressions.

## INTRODUCTION

Language use in conversation requires conversation partners to consider each other’s points-of-view, or perspectives. This is necessary for successful communication in contexts where participant information states differ, either because they hold different visual perspectives on the scene, or differing beliefs. Beyond this, some grammatical phenomena in natural language are inherently perspectival: their meaning depends on a semantically represented perspective-holder. This paper explores the production and interpretation of such grammatically perspectival expressions, focusing in particular on the perspectival motion verb *come* in English.

A large body of existing work has explored how conversation partners take into account differences in their information states, often referred to as their ‘perspective.’ However, relatively little work has looked at the processes involved in grammatically perspectival expressions, which encode reference to point-of-view in their semantics. When an expression must be interpreted relative to a particular perspective, how do speakers decide whose perspective to use? How to listeners infer the perspective that is being used when interpreting a grammatically perspectival expression?

Early work on perspective-taking in conversation suggested that speakers often fail to take into account differences between their visual perspective and that of their listener when choosing referring expressions (Epley, Keysar, et al., 2004; Epley, Morewedge, & Keysar, 2004; Keysar et al., 2000, 2003). These communicative failures were explained as a result of **egocentricity**: a cognitive bias towards self perspectives (Epley, Keysar, et al., 2004; Keysar et al., 2000). However, more recent work suggests that conversation partners do take into account information asymmetries in both production and comprehension (Bezuidenhout, 2013; Brown-Schmidt & Hanna, 2011; Hanna et al., 2003; Hawkins et al., 2021; Heller et al., 2008, 2016; Kuhlen & Brennan, 2013; Ryskin et al., 2020).

In this paper, we re-visit the question of egocentric bias in a less well-studied domain of perspective-taking: the production and comprehension of grammatically perspectival expressions. We lay out various models of perspective inference and selection for perspectival expressions (Anderson & Dillon, 2019; Harris, 2012; Kuno & Kaburaki, 1977), focusing in particular on two models of grammatical perspective-taking: a serial model, the speaker anchoring-and-adjustment model; and a parallel model, the simultaneous integration model (Modeling Grammatical Perspective-Taking section).

We test the differing predictions of these models in a series of comprehension and production experiments using the perspectival motion verb *come* as a case study. To foreshadow our results, our comprehension studies (Perspective Inference in Comprehension section and Comprehension Experiments section) suggest that listeners reason simultaneously over multiple perspectives, as in the simultaneous integration model proposed by Anderson and Dillon (2019), consistent with Heller et al. (2016) and Ryskin et al. (2020)’s analysis of perspective-taking in reference. However, our production findings (Perspective Selection in Production section and Production Experiments section) are more mixed: we find support for only one of the simultaneous integration model’s two key predictions.

Although our results are largely compatible with a simultaneous integration model of grammatical perspective-taking, the attested asymmetry between production and comprehension is problematic for a strongly Bayesian view of conversation, where speakers and listeners iteratively finetune their models of each others’ behavior. We conclude with a discussion of how our grammatical perspective-taking findings fit into the broader picture of perspective-taking in conversation and rational approaches to conversation (General Discussion section).

## PERSPECTIVE-TAKING AND EGOCENTRICITY

Egocentric biases in conversation have been most thoroughly studied in reference. How do conversation participants choose to refer to objects when their partners’ knowledge of the object might be different than their own? A common way of manipulating the information states of conversation participants is to set up different visual perspectives on a scene. One common experimental paradigm involves pairs of participants who sit on opposite sides of a display of boxes such that the contents of some of the boxes are hidden from one participant (Brown-Schmidt et al., 2008; Hanna et al., 2003; Keysar et al., 2000; Nadig & Sedivy, 2002; Rubio-Fernández, 2017), setting up an information asymmetry between the two. The extent to which a speaker considers their listener’s information state then can be measured by comparing how they refer to the visible and obscured objects.

**Figure 1.**
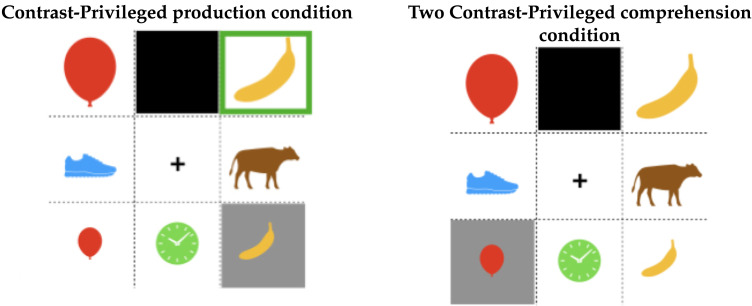
Production and comprehension stimuli from Ryskin et al. (2020).

Figure 1 shows two examples of displays from an experiment using this paradigm reported in Ryskin et al. (2020). The black boxes represent boxes that are occluded from the view of one participant. When shown the display pictured on the left, a speaker might produce *The big banana* to describe the highlighted referent. However, if they consider that only one banana is visible to the listener, they might decide the modifier is unnecessary and produce *The banana*. The rate of (unnecessary) modification therefore measures the speaker’s bias towards their own information state (perspective).

In the comprehension version of the task, listeners’ awareness of information asymmetries can be measured through their reaction times for selecting a referent. The right image in Figure 1 shows a paradigm with two size contrasts. If the listener considers that the second balloon is hidden from the speaker, they can infer that the intended referent is the large banana as soon as they hear *Click on the big*…. But if they fail to take into account the speaker’s visual perspective, they may wait to hear whether the next word is *banana* or *balloon*.

Early work using this paradigm found that speakers often failed to consider the information asymmetries caused by differing visual perspectives (Epley, Keysar, et al., 2004; Epley, Morewedge, & Keysar, 2004; Keysar et al., 2000, 2003; Lin et al., 2010). These findings motivated the proposal of a general cognitive bias towards self-perspectives known as **egocentric bias**. The intuition is that conversation partners start out using their own perspectives, but switch to consider their partners’ perspectives when necessary. In this view, accessing self-perspectives is automatic, but access to other perspectives comes at a cognitive cost (Lin et al., 2010; Todd & Simpson, 2016; Wardlow, 2013). This is known as the **egocentric anchoring-and-adjustment** model of perspective-taking (Epley, Keysar, et al., 2004).

More recent work has called the strength of egocentricity into question (Bezuidenhout, 2013; Brown-Schmidt & Hanna, 2011; Hanna et al., 2003; Hawkins et al., 2021; Heller et al., 2008, 2016; Kuhlen & Brennan, 2013; Mozuraitis et al., 2018; Ryskin et al., 2020). After accounting for potential experimental design confounds in earlier visual paradigms (Bezuidenhout, 2013; Kuhlen & Brennan, 2013), subsequent work has found that speakers and listeners generally do consider each other’s perspectives when producing and interpreting referring expressions (Hawkins et al., 2021; Heller et al., 2016). This more recent line of work proposes that perspective-taking in reference involves **simultaneous integration of multiple perspectives**: conversation partners take into account both their own perspective and their partner’s information state. In this model, referring expressions are produced and interpreted according to a weighted balance of the egocentric and shared perspective on the scene.

Visual perspective-taking is not the only domain in which information asymmetries between speakers and listeners have been studied. Other work on information asymmetries and referring expressions has used mismatches in speaker-listener knowledge about the name or function of an object (Isaacs & Clark, 1987; Mozuraitis et al., 2015, 2018). For instance, Mozuraitis et al. (2018) find that speakers modify how they refer to objects based on whether they think the listener knows its function. Bergen and Grodner (2012) show that listeners consider the speaker’s likely knowledge state in processing scalar implicatures even in contexts where the speaker is not present or known. There is also work on spatial deixis in dyadic communication, though most prior work has focused on understanding the semantics of deictic terms (Rubio-Fernández, 2021; Shin et al., 2020; Skilton & Peeters, 2021).

Although there is ongoing debate over the strength of egocentric biases in reference perspective-taking, the notion of egocentric bias has been influential across domains. This paper explores whether egocentricity effects observed in reference generalize to a domain that is relatively underexplored in experimental work: the selection and inference of perspective-holders for grammatically perspectival expressions.

## GRAMMATICAL PERSPECTIVE

Perspectival expressions constitute a diverse class of phenomena. A common property of these expressions is that a component of their meaning refers relative to the perspective of an individual. Some perspectival expressions convey the individual tastes or preferences of their perspective-holder, such as predicates of personal taste (*tasty*). Others communicate their beliefs or attitudes, such as expressives (*damn*) and epithets (*that jerk*). Still others refer relative to the perspective-holder’s location (*come*) or position (*right*).

For instance, the perspectival motion verb *come* conveys motion towards a particular individual in the discourse, referred to as the **perspective-holder**. Although the perspective-holder is often the speaker, it need not be. For instance, in (1), the perspective-holder is the speaker, while in (2), motion is directed towards the listener.

1.  You are coming to my house tonight, Penelope.
2.  I am coming to your house tonight, Penelope.

Not all individuals can serve as perspective-holders; broadly speaking, a perspective-holder must be a prominent individual in the discourse. All conversation participants are potential perspective-holders, since they are always important to the discourse. In English, other common perspective-holders include subjects of attitude verbs and protagonists of narratives.^1^ For instance, in (3), the perspective-holder is Frodo, because he is the protagonist of the novel.

3.  In the second chapter of *The Fellowship of the Ring*, Gandalf comes to warn Frodo about the ring.

The factors that determine perspective-holder prominence are the subject of ongoing investigation. A body of work on predicates of personal taste (e.g., *tasty*) suggests that thematic roles are important (Kaiser, 2020; Kaiser & Lee, 2017a, 2017b). Work on Free Indirect Discourse, a kind of perspective shift environment, suggests that both global discourse factors, like topicality and coherence relations, and local discourse factors, like argument structure and thematic roles, play a role in determining who can serve as the perspective-holder (Abrusán, 2021; Bimpikou, 2020; Harris, 2012; Hinterwimmer, 2019; Kaiser, 2015; Meuser et al., 2020).

It is also an open question whether various classes of perspectival expressions share a common semantics. Here we focus on one sub-class of perspectival expressions to investigate the mechanisms underlying grammatical perspective-taking: the perspectival motion verbs *come* and *go*.

### Perspectival Motion Verbs

**Figure 2.**
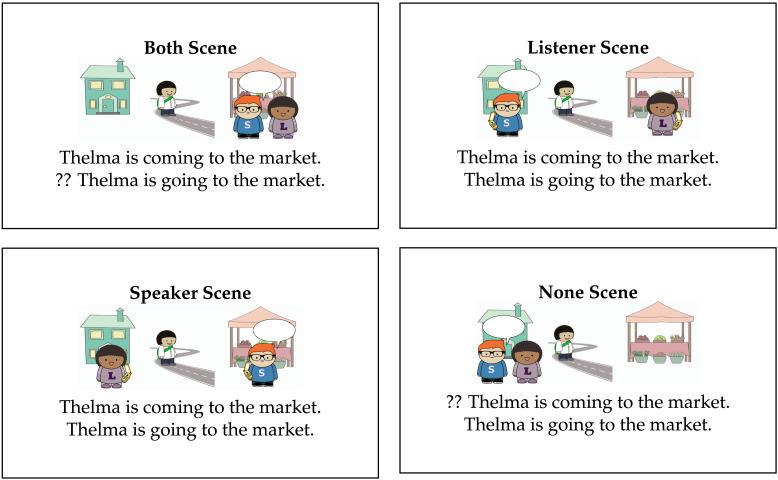
Acceptability of *come* and *go* in different contexts.

Perspectival motion verbs describe motion relative to a perspective-holder. The perspectival motion verb *come* describes motion towards the location of the perspective-holder. As illustrated in Figure 2, *come* can be felicitously used to describe motion towards either the speaker’s or listener’s location. However, it cannot be used felicitously in the context shown in the None scene, since there is no individual at the destination of motion to serve as the perspective-holder.

In contrast, the motion verb *go* describes motion that is not towards the location of the perspective-holder. It is therefore felicitous as a description of the None scene, since no potential perspective-holder is located at the destination. It can also be used to describe the Listener scene, if the speaker is the perspective-holder, or the Speaker scene, if the listener is the perspective-holder. What about the Both scene? This world cannot be felicitously described using *go*, because all potential perspective-holders in this context are at the destination of the motion.

Formal analyses of the semantics of *come* treat its perspectival component differently (Charnavel, 2018; Fillmore, 1966; Goddard, 1997; Oshima, 2006a, 2006b; Sudo, 2018; Taylor, 1988). For concreteness, we adopt a perspective-anaphoric treatment based on the arguments presented in Barlew (2017). In this analysis, *come* is anaphoric to a prominent perspective in the Common Ground.

4.  **Lexical semantics of** *come*: [[*come*(*x*, *d*)]]^*w*,*a*^ = T iff
  (a)  Motion implication:
      [[∃*e*.move(*x*, *e*) ∧ dest(*d*, *e*)]]^*w*,*a*^ = T
  (b)  Anchoring implication:
      ∃*y*.[[loc(*y*, *d*)]]^*w*,*a*^ = T and *y* is a prominent individual holding perspective *a*
      Where *e* is an event, *d* is a location, *w* is a world, *x* and *y* are an individuals, and *a* is a perspective.
5.  **Lexical semantics of** *go*: [[*go*(*x*, *d*)]]^*w*,*a*^ = T iff
  (a)  Motion implication:
      [[∃*e*.move(*x*, *e*) ∧ dest(*d*, *e*)]]^*w*,*a*^ = T
  (b)  Anchoring implication:
     ∃*y*.[[loc(*y*, *d*)]]^*w*,*a*^ = F and *y* is a prominent individual holding perspective *a*
      Where *e* is an event, *d* is a location, *w* is a world, *x* and *y* are an individuals, and *a* is a perspective.

We use *a* to represent variables that range over perspectives, and assume that there is an assignment function that maps such variables to perspectives prominent in the Common Ground.

Accordingly, perspective resolution parallels other sorts of anaphoric expressions, such as pronouns. From a production standpoint, the speaker’s goal is to select a compatible perspective-holder and a motion verb to describe the intended motion event. From a comprehension standpoint, the listener’s goal is to infer the speaker’s adopted perspective and intended meaning from their utterance. Although we have adopted Barlew (2017)’s anaphoric analysis for concreteness, this characterization remains the same under competing analyses of *come*, insofar as they all involve uncertainty over the identity of the perspective-holder.

Whether or not *go* is lexically specified as perspectival is an open question. Some analyses treat *go* as lexically restricted against motion towards the perspective-holder, making *go* ungrammatical in the Both scene (Oshima, 2006a). However, others have proposed that *go* is infelicitous there because of pragmatic competition with *come* (Sudo, 2018; Wilkins & Hill, 1995), which leads to an anti-perspectival inference when *go* is used. The modeling work presented in Anderson and Dillon (2019) suggests that both views are viable and empirically difficult to distinguish. We use a perspectival semantics for go, though our core predictions do not rest on it.

Although perspectival motion verbs are the subject of significant cross-linguistic comparative work (Barlew, 2017; Gathercole, 1987; Nakazawa, 2007, 2009; Wilkins & Hill, 1995), there is relatively little experimental work on them. This gap leaves open many questions about their usage, and relates to models of grammatical perspective-taking. We turn now to these models.

### Egocentricity in Grammatical Perspective-Taking

Although perspectival expressions are a diverse category, one of their shared characteristics is a tendency to be interpreted relative to the speaker’s perspective (Fillmore, 1966; Harris & Potts, 2009; Kuno & Kaburaki, 1977; Lasersohn, 2005; Potts, 2005, 2007). There is a growing body of work focused on quantifying the strength of this preference for speaker perspectives in grammatical perspective-taking (Harris, 2012; Harris & Potts, 2009; Kaiser, 2015; Watson et al., 2021), but its source remains a topic of debate.

The preference for speaker perspectives cannot be hard-coded into the grammar of perspectival expressions, since the set of perspective-holders is contextually determined (Barlew, 2017; Harris, 2012). Consequently, some work has attempted to encode it via a rule-based approach to perspective selection (Kuno & Kaburaki, 1977) or via obligatory grammatical processes (Speas & Tenny, 2003).

More recent work takes inspiration from models proposed for reference perspective-taking and posits that the preference is a result of egocentric bias (Anderson & Dillon, 2019; Harris, 2012; Watson et al., 2021). In this view, there is a general cognitive bias towards self perspectives, which causes speakers to preferentially use their own perspectives when producing perspectival expressions.

Appealing to a general cognitive bias is attractive, because it offers a unified explanation for effects in the reference perspective-taking and grammatical perspective-taking domains. However, there are a number of differences between the two domains that may complicate attempts to extrapolate from what is known about perspective-taking in reference to grammatical perspective-taking.

First, work on perspective-taking in reference explores information asymmetries between the speaker and listener, whether caused by differences in participants’ visual viewpoints (Heller et al., 2016; Ryskin et al., 2020), or by differences in knowledge about an object visible to both participants (Mozuraitis et al., 2015, 2018). Differences in grammatical perspective, on the other hand, can arise even if all conversation participants are in the same information state.

Second, the expressions investigated in reference perspective-taking are not grammatically perspectival: they consist of simple referring expressions. In grammatical perspective-taking, the semantics of the expressions of interest contain a free perspectival variable whose referent is contextually determined.^2^ Thus, even if the speaker and listener have identical information, the identity of the perspective-holder may be ambiguous, in the same way that the antecedent of a pronoun may be ambiguous.

Lastly, perspectival expressions have a unique perspective-holder. In reference perspective-taking, it is possible to integrate the knowledge states of the speaker and listener to varying degrees: we can construct a Common Ground perspective consisting only of objects visible to both participants. In grammatical perspective-taking, however, the perspective-holder must be identified with a unique individual. If the speaker is in Seattle and the listener is in Boston, the perspective-holder is either on one coast or the other; there is no potential Common Ground perspective-holder in the Midwest.

## MODELING GRAMMATICAL PERSPECTIVE-TAKING

Because of the differences between reference perspective-taking and grammatical perspective-taking, we do not expect the two domains to involve identical processes and biases. However, the models and frameworks from perspective-taking in reference can help guide our thinking about the production and comprehension of grammatically perspectival expressions. In this section, we lay out two models of grammatical perspective-taking that draw on prior models of reference perspective-taking (Epley, Keysar, et al., 2004; Heller et al., 2016; Keysar et al., 2000; Mozuraitis et al., 2018; Ryskin et al., 2020) and theoretical work on grammatical perspective-taking (Harris, 2012; Kuno & Kaburaki, 1977): a serial model that encodes a strong bias towards the speaker’s perspective, and a parallel model in which multiple perspectives are considered simultaneously.

### Anchoring-and-Adjustment Models

One hypothesis about grammatical perspective-taking is that it is governed by a hierarchy of perspective-holders. Kuno and Kaburaki (1977) propose an analysis of come where its perspective-holder is determined according to an **empathy hierarchy**: the availability of a perspective-holder depends on the degree that the speaker identifies with the individual.

6.  **Speech-Act Empathy Hierarchy** (Kuno & Kaburaki, 1977):
  (a)  The speaker empathizes most strongly with themselves.
  (b)  The speaker empathizes more closely with the listener than third persons.
  (c)  The speaker empathizes most with the subject of the sentence.
  (d)  After the subject, the object is the easiest to empathize with.
  (e)  Objects of passive by-agentive clauses are the hardest to empathize with.

Although not proposed as an explicit processing model of perspective-taking, the empathy-based approach to perspective prominence bears a strong similarity to egocentric anchoring-and-adjustment models of reference perspective-taking (Epley, Keysar, et al., 2004; Keysar et al., 2000), since it assumes a serial model of perspective-taking. In both views, there is a default perspective that the speaker always selects, and the speaker switches to another perspective when the default perspective is unavailable or infelicitous.

If we view Kuno’s empathy hierarchy as a set of perspective selection and inference heuristics, we can adapt them into a model of grammatical perspective-taking. The result would be a serial model of grammatical perspective-taking, in which conversation participants first adopt the perspective that they empathize most closely with. If this perspective is incompatible with the given context, then they select another perspective, according to the empathy hierarchy: the speaker, then the listener, then third-persons based on their grammatical role.

In production, this serial model predicts a strong preference for speaker perspectives: speakers should use their own perspective whenever possible, since they empathize most closely with themselves.

For comprehension, there is a choice to be made. If egocentricity is a general cognitive bias, then we would expect it to influence listeners as well as speakers. If so, listeners should first interpret a perspectival expression according to their own perspective. If the resulting interpretation is infelicitous, they would then switch to the speaker’s perspective. This is similar to the speaker anchoring-and-adjustment model of perspective-taking put forward in the reference domain in Epley, Keysar, et al. (2004) and Keysar et al. (2000).

Alternatively, we could treat egocentricity as a kind of guiding heuristic, rather than a strong cognitive bias. In this case, listeners might actually anticipate a speaker’s own egocentric bias, and use it to guide their interpretation. In this model, listeners will select the speaker’s perspective first, and use their own only if the speaker’s is incompatible with the context (i.e., the sentence would be infelicitous).

Which of these interpretations of egocentricity is most promising for the grammatical perspective-taking domain? We draw here on the existing experimental work on grammatical perspective-taking, which finds a strong preference for speaker-oriented interpretations of epithets, expressives, and predicates of personal taste in comprehension (Harris, 2012; Kaiser, 2015; Kaiser & Lee, 2017a, 2017b). The data from these comprehension experiments suggest that comprehenders are guided by awareness of the speaker’s self-bias.

These findings lead Harris (2012) to adopt the latter view of egocentricity. He proposes a two-stage model of perspective inference, where listeners have both a complex perspective reasoning system and a simpler heuristic-based system. In the simpler system, listeners assume that by default, speakers use their own perspectives.^3^ When necessary, listeners can switch to the more complex perspective reasoning system and take multiple perspectives into consideration. This switch is triggered when the sentence would be infelicitous according to the speaker’s perspective.

Drawing on Keysar et al. (2000) and Epley, Keysar, et al. (2004)’s model of perspective-taking in reference, as well as Harris (2012)’s approach to speaker bias, we call this a **speaker anchoring-and-adjustment model** of grammatical perspective-taking: both speakers and listeners consider the speaker’s perspective by default. In the speaker anchoring-and-adjustment model, the speaker’s perspective is the default in both comprehension and production. Comprehenders will first attempt to interpret perspectival expressions according to the speaker’s perspective, and speakers will produce expressions according to their own perspectives, unless the context eliminates the speaker’s perspective.

### Simultaneous Integration

A competing hypothesis about grammatical perspective-taking is that conversation partners reason about multiple perspectives simultaneously. In the simultaneous integration view of perspective-taking, speakers and listeners are aware that their perspectives may differ from that of their partner, and use their awareness of multiple perspectives to guide production and comprehension.

Anderson and Dillon (2019) propose a simultaneous integration model of grammatical perspective-taking formulated in the Rational Speech Acts framework. In this model, listeners probabilistically reason jointly about the speaker’s intended meaning and their adopted perspective using a mental model of the speaker’s production process. Although Anderson and Dillon (2019) propose a model for comprehension only, we extend their Perspectival Rational Speech Acts model to the production of grammatically perspectival expressions as well.

#### Comprehension.

In this simultaneous integration model, the listener’s task is to simultaneously infer the speaker’s intended meaning and their adopted perspective. Rather than selecting a single perspective (e.g., the speaker’s) and attempting to interpret the speaker’s utterance, as in the speaker anchoring-and-adjustment model, the listener reasons jointly over all perspective-meaning pairs. As a result, the listener takes into account all possible perspectives jointly during inference. To settle on the speaker’s intended meaning, the listener then marginalizes over all possible perspectives.

This results in a simultaneous consideration of all perspectives, as in the simultaneous integration model for reference perspective-taking proposed in Hawkins et al. (2021), Heller et al. (2016), and Ryskin et al. (2020). In the Anderson and Dillon (2019) model, like in the Hawkins et al. (2021) model of reference perspective-taking, the integration of the multiple perspectives is done through joint Bayesian inference rather than by calculating a weighted combination of perspectives, as in the Ryskin et al. (2020) model.

#### Production.

In production, the speaker’s goal is to select the best utterance-perspective pair to communicate their intended meaning, which they do by reasoning about how the listener will interpret each candidate utterance. Because the speaker cannot directly communicate the adopted perspective to the listener, they must consider how the listener will interpret an utterance according to each potential perspective that the listener might adopt. The most useful utterance is the one that is most likely to be understood by the listener under all possible perspectives. As in the simultaneous integration model for reference perspective-taking, however, the listener may be more likely to adopt some perspectives than others. The speaker takes this into account by setting a prior probability on each perspective in their model of the listener.

Although the speaker jointly calculates the probabilities of utterance-perspective pairs, they ultimately select a single utterance to produce by marginalizing over all perspectives. This results in a simultaneous integration model: the speaker’s choice of utterance takes into account its interpretation according to all perspectives.

#### Iterated Recursive Reasoning.

The Anderson and Dillon (2019) model employs recursive Bayesian reasoning. The actual listener is represented by a model called the **Pragmatic Listener**. Given an utterance *u* from the speaker, the Pragmatic Listener’s goal is to estimate the joint probability of the speaker’s intended meaning *w*, modeled as a **possible world** they are describing, and the speaker’s adopted perspective *a*. For each possible meaning that the speaker could be trying to communicate, the listener’s goal is to estimate its likelihood given the speaker’s utterance. However, the meaning of an utterance containing a perspectival expression depends on the perspective that has been adopted. Since the speaker’s choice of perspective is not directly observable, the listener tries to estimate the probability of meaning-perspective pairs (*p*(*w*, *a*|*u*)), and then **marginalizes** over the set of possible perspectives to find the most likely meaning (max*_w_ p*(*w*|*u*)).

Their calculation takes into account two things: how likely they think the possible world is (*p*(*w*)), and how likely they think the speaker is use the given utterance to express that particular world-perspective pair (*p*(*u*|*w*, *a*)). The listener estimates the prior probability of the meaning according to their own set of beliefs about the world. They estimate the second component by relying on a mental model of the speaker’s production process: the **Pragmatic Speaker**.

The Pragmatic Speaker’s goal is to select a perspective-utterance pair to express their intended meaning *w*. That is, they are reasoning jointly over the utterance/perspective pair that is most likely to communicate *w* successfully to the listener: their goal is to calculate the likelihood that each utterance/perspective pair will lead to success (*p*(*u*, *a*|*w*)). To do this, they take into account the likelihood of the listener understanding their utterance (*p*(*w*|*u*, *a*)), as estimated by running a simplified model of the listener called the **Literal Listener**. They also take into account the prior probability of the perspective (*p*(*a*)), and of the utterance given the perspective (*p*(*u*|*a*)).

The Literal Listener is the speaker’s simplified mental model of the listener. Its task is to infer the probability of a world given a perspective-utterance pair, *p*(*w*|*u*, *a*), which it does by evaluating the utterance according to the world and perspective, and taking into account the prior probability of the world and the perspective. The Literal Listener does not correspond to a real-world listener, since perspectives are not usually directly observable.

This model was originally proposed by Anderson and Dillon (2019) as a concrete implementation of Harris (2012)’s second-stage abductive reasoning system for grammatical perspective-taking in comprehension. We extend the Anderson and Dillon (2019) model to production by adding an additional Pragmatic Speaker level to model the actual speaker. This allows us to model a speaker who reasons about their listener’s perspective inference process using the Pragmatic Listener as its mental model of the listener. As a result, our extended PRSA model is more similar to Hawkins et al. (2021)’s fully Bayesian model of reference perspective-taking than to the simultaneous integration model proposed in Heller et al. (2016) and Ryskin et al. (2020), in which only the listener engages in recursive Bayesian reasoning.

#### Perspective Cost.

In the Anderson and Dillon (2019) model, both the speaker and listener are aware that the speaker may be biased towards their own perspectives. The speaker’s egocentric bias is incorporated through the use of a perspective cost function. This cost function penalizes non-speaker perspectives in the model of the speaker. A higher perspective cost setting results in speaker behavior that is more egocentric; a perspective cost of zero results in equal weighting of all perspectives.

We note that the perspective cost also affects the listener. Because the Pragmatic Listener’s calculation relies on the Pragmatic Speaker, manipulating the perspective cost affects the listener’s behavior as well as the speaker’s. As the perspective cost increases, comprehenders are more likely to assume that the speaker is using their own perspective.

#### Extended Perspectival Rational Speech Acts model.

**Figure 3.**
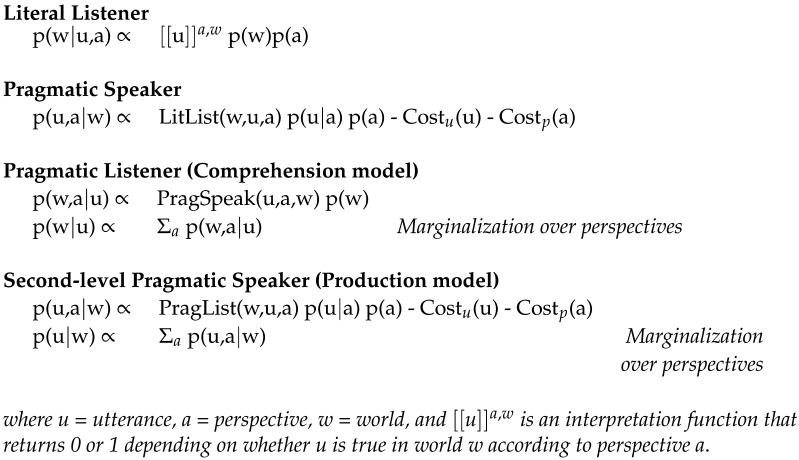
Perspectival Rational Speech Acts model (revised).

The resulting Perspectival Rational Speech Acts model for comprehension and production is expressed formally in Figure 3. We show the joint inference equation as well as the final marginalization step which the listener uses to settle on a single interpretation and the speaker uses to select an utterance to produce.

The PRSA model achieves the same kind of perspective-mixing assumed in the Heller et al. (2016), Mozuraitis et al. (2018), and Ryskin et al. (2020) simultaneous integration model of reference perspective-taking and the Watson et al. (2021) model of grammatical perspective-taking, but through different mathematical means. Their model uses an explicit perspective-mixing parameter to explore bias towards a particular perspective. By default, the PRSA assumes equal weight on all perspectives due to the marginalization operation; however, perspective bias can be explored by manipulating the perspective cost function.

### Summary

The speaker anchoring-and-adjustment and simultaneous integration models of grammatical perspective-taking represent two different ways of thinking about perspective inference and selection. In the speaker anchoring-and-adjustment model, conversation participants select one perspective at a time, and only reconsider their choice if the given perspective is not compatible with the discourse context. In the simultaneous integration model, by contrast, conversation participants consider multiple perspectives at once. Each incorporates an egocentric bias in production: in the speaker anchoring-and-adjustment model, the speaker’s perspective is always selected first, while in the simultaneous integration model, non-speaker perspectives are more costly for the speaker to adopt. The different assumptions that each model makes about how conversation participants select perspectives lead to different predictions about how conversation participants will use and interpret grammatically perspectival expressions.

These two models correspond to the two systems for perspective inference posited by Harris (2012). The speaker anchoring-and-adjustment model is a heuristic-based system, which may fail, causing the listener to re-sample a perspective and try again. This corresponds to the simpler first-stage model. The simultaneous integration model corresponds to the second, more complex system that listeners have in Harris (2012)’s proposal. We take the mathematical model for the latter comprehension system from Anderson and Dillon (2019) and extend it to the production of grammatically perspectival expressions.

Integrating the speaker anchoring-and-adjustment and simultaneous integration models for comprehension corresponds to Harris (2012)’s proposed two-stage model of comprehension, with some sort of contextual evidence providing a cue to switch between the two.^4^ Adopting only the speaker anchoring-and-adjustment model for both comprehension and production would parallel the theoretical treatment of perspective proposed in Kuno and Kaburaki (1977). Combining the speaker anchoring-and-adjustment model of production with the simultaneous integration model of comprehension would lead to a model similar to the one that Kehler and Rohde (2013) propose for pronominalization, in which listeners consider more sources of evidence than speakers.

Having outlined two competing models of grammatical perspective-taking for both comprehension and production, we turn to testing their predictions about perspective inference in comprehension and perspective selection in production. In Perspective Inference in Comprehension section and Comprehension Experiments section, we explore their predictions for grammatical perspective-taking in comprehension, and present three comprehension experiments that test a key difference between them. In Perspective Selection in Production section and Production Experiments section, we discuss the predictions that each model makes for production, and provide empirical evidence about perspective selection in two key contexts.

The predictions that we present are generated using computational implementations in the WebPPL probabilistic programming language. For the simultaneous integration model, we generate predictions using perspective costs ranging from 0 (no speaker bias) to 1 (strong speaker bias). Our code can viewed through the Open Science Foundation.^5^

## PERSPECTIVE INFERENCE IN COMPREHENSION

In comprehension, listeners must infer the speaker’s intended meaning and their adopted perspective. This section explores the predictions that the speaker anchoring-and-adjustment and the simultaneous integration models make for grammatical perspective-taking in comprehension.

**Figure 4.**
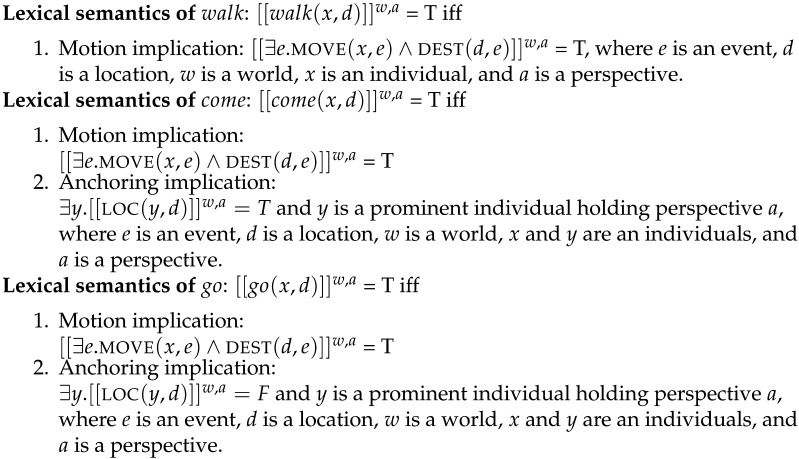
Lexical semantics for candidate verbs.

We look at model predictions for three sentence frames: *X is going to the market*, *X is coming to the market*, and *X is walking to the market*. The lexical semantics for the verbs are shown in Figure 4. Note that it follows from the semantics of *come* that the subject cannot be the perspective-holder, since it is impossible to be simultaneously in motion towards a place and already located at that place.

We use these semantics for all models; models differ only in their perspective selection and inference mechanisms. We assume the same interpretation function for all models: given a world (utterance context) and a perspective, the interpretation function maps an utterance to a truth-value according to the lexical semantics in Figure 4.

**Figure 5.**
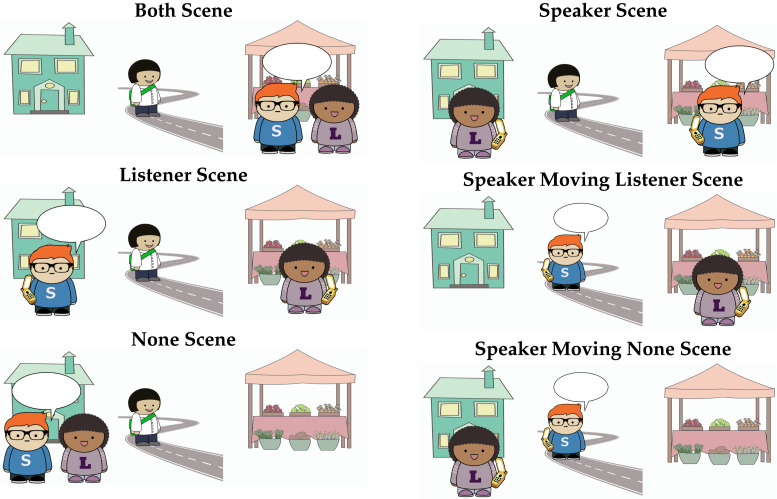
**Set of possible scenes with two locations, the house and the market, and three referents.** In all scenes, Speaker Sam is marked with an S and Listener Lucy is marked with an L. Thelma Third Person is unmarked.

In what follows, we represent utterance contexts with illustrated scenes. We consider a set of perspective-taking scenarios with two locations (here, the house and the market) and three individuals: a speaker, Sam, a listener, Lucy, and a third person, Thelma, whose perspective is not accessible. We focus on the set of six scenes presented in Figure 5. We include four scenes where Thelma is the mover and two where the speaker, Sam, is the mover. When Sam is the mover, Thelma is absent from the scene in order to make it clear that she is not a potential perspective-holder.

### Speaker Anchoring-and-Adjustment Comprehension Predictions

Recall that in the speaker anchoring-and-adjustment model, listeners first try to interpret grammatically perspectival expressions according to the speaker’s own perspective, and if this interpretation fails, the listener selects a different perspective and tries again.

This model predicts that comprehenders should initially expect the speaker to be located at the destination of motion when presented with an utterance containing *come*, since they expect the speaker to be the perspective-holder, and *come* requires the perspective-holder to be at the destination of motion. Out of the six scenes we consider, only the Both and Speaker scenes satisfy this expectation. Crucially, this model predicts no difference between these two scenes, since the listener’s perspective is not considered when the speaker’s is available.

If the speaker-oriented interpretation fails, the listener will re-analyze according to another perspective: their own. This leads them to expect the listener to be at the destination of motion (Listener, Speaker Moving Listener). The Speaker Moving Listener and Listener conditions differ only in when the speaker-oriented interpretation is predicted to fail: in the Speaker Moving Listener condition, unlike the Listener condition, the utterance itself (*I am coming*) indicates that the speaker is not using their own perspective. Use of *come* when no one is at the destination is predicted to be ungrammatical (None, Speaker Moving None).

Thus, the speaker anchoring-and-adjustment model predicts a **Simple Speaker Advantage** for utterances with *come*: all scenes where the speaker is at the destination are equally likely, and more likely than any others. Given an utterance *Thelma is coming* …, the listener should rank the scenes in likelihood as follows: {Speaker, Both} > {Listener} > {None}. Given an utterance *I am coming* …, they should rank the possible scenes in likelihood as follows: {Speaker Moving Listener} > {Speaker Moving None}. However, the second utterance is surprising in this model: listeners will expect the speaker to produce *I am going* or *I am walking*, since they are compatible with the speaker’s perspective.

### Simultaneous Integration Comprehension Predictions

The simultaneous integration model of comprehension posits that listeners consider multiple possible perspective-holders at once. As a result, the simultaneous integration model predicts that *come* is most likely to describe a scene where multiple potential perspective-holders are at the destination of motion. This follows from the listener’s consideration of the speaker’s production process: *come* is most useful when it is a valid description of the intended scene according to all potential perspectives.

We call this the **Convergent Perspective Boost**: the Both scene receives a boost in probability when the perspectives of the potential perspective-holders converge, because the scene is compatible with multiple perspective-holders (both speaker and listener). Because of this effect, the simultaneous integration model predicts that when listeners hear *Thelma is coming* …, they will place the highest probability on the scene in which both potential perspective-holders are at the destination of motion (Both).

The scenes in which only one candidate perspective-holder is at the destination will also be somewhat likely (Speaker, Listener); their relative probability depends on the perspective cost function setting. Use of *come* when no one is at the destination is predicted to be unlikely, since it is ungrammatical (None, Speaker Moving None).

The model therefore predicts that given an utterance *Thelma is coming* …, the listener should rank the scenes in likelihood as follows: {Both} > {Speaker} > {Listener} > {None}, assuming that there is a non-zero perspective cost.

Unlike in the speaker anchoring-and-adjustment model, listeners should not be surprised by the speaker producing *I am coming* …; in this case, they should infer with high probability that the speaker is describing the Speaker Moving Listener scene. In fact, since the perspective-holder is unambiguous when the speaker is the subject of the motion verb, *I am coming* … should be easier for listeners to interpret than *Thelma is coming* …, since they can infer with certainty that the scene is the Speaker Moving Listener one, rather than spreading some probability across the Both, Speaker, and Listener scenes.

### Summary

The speaker anchoring-and-adjustment and simultaneous integration models of comprehension make different predictions about perspective inference in comprehension when a third character is moving. The critical difference lies in the predicted relative probability of the Both and Speaker scenes. The speaker anchoring-and-adjustment model considers only one perspective at a time, and essentially reduces them to the same scenario: both scenes benefit from the Simple Speaker Advantage. The simultaneous integration model, by contrast, predicts an advantage for the Both scene over the Speaker scene due to the Convergent Perspective Boost: *come* is felicitous in this scene according to either candidate perspective-holder. We test this key difference in the predictions of the two models in a series of comprehension experiments.

## COMPREHENSION EXPERIMENTS

We ran three experiments to explore a key difference between the predictions of the two models. As detailed above, the simultaneous integration model critically predicts a Convergent Perspective Boost: the most likely scene to be described by *Thelma is coming* is the Both scene. By contrast, the speaker anchoring-and-adjustment model predicts a Simple Speaker Advantage: all scenes where the speaker is at the destination should be equally likely, because they are consistent with the first-selected perspective, that of the speaker.

**Figure 6.**
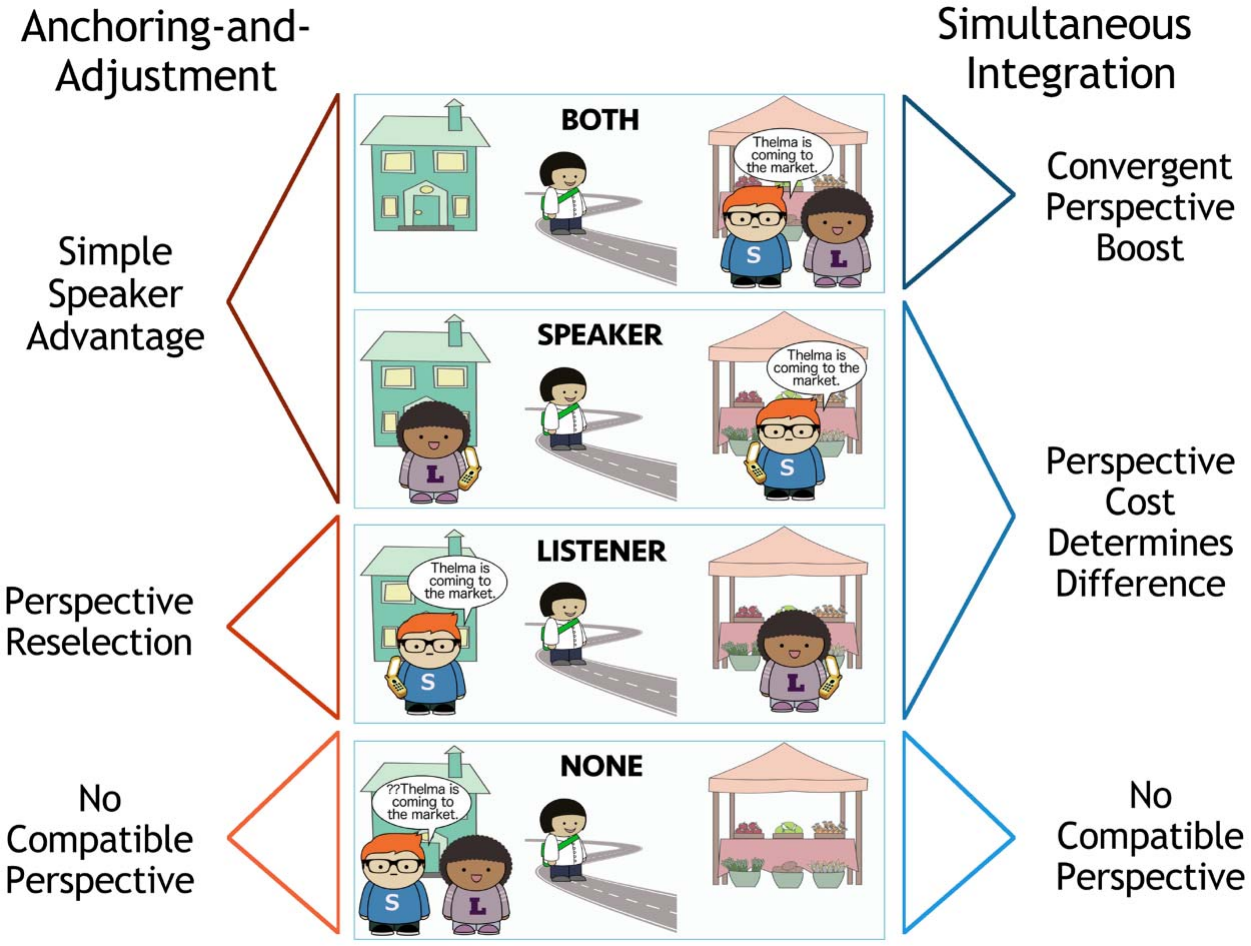
Ranked likelihood of scenes given *Thelma is coming to the market* as ranked by Convergent Perspective Boost versus Simple Speaker Advantage.

We summarize the predictions of the two models in Figure 6. When a listener hears *Thelma is coming*, according to the speaker anchoring-and-adjustment model, they should find the Both and Speaker scenes equally, and the Listener scene less likely, but possible. According to the simultaneous integration model, they should find the Both scene most likely, followed by the Speaker scene, and then the Listener scene, with the relative likelihood of the last two depending on the strength of the perspective cost.

We test these predictions in a series of paired sentence/picture compatibility judgment tasks. We use illustrated scenes to represent an observed world (meaning) that the speaker seeks to communicate. In our paradigm, participants first saw a sentence being spoken by a character, and then a scene depicting an utterance context for the sentence. Their task was to judge whether the scene and sentence were compatible.

In this paradigm, our linking hypothesis is that participants should be fastest to accept scenes when they depict worlds that the listener thinks the speaker is likely to be describing with the given utterance (i.e., worlds with high marginal posterior probability in the listener’s comprehension model). Somewhat more formally, we suppose that reaction time in this task is monotonically related to the surprisal (negative log probability) of a world given a sentence (following Nordmeyer and Frank (2014)).

Materials, data, and analysis scripts for all experiments are hosted by the Open Science Foundation.^6^

### Experiment 1a

Experiment 1a is a paired sentence/picture compatibility judgment task exploring the relative compatibility of the Speaker and Both scenes with *come*.

#### Methods

##### Participants.

128 monolingual American English-speaking participants were recruited through Prolific. Participants who achieved less than 90% accuracy on a spatial control task (described below) were excluded from the experiment (*n* = 48), leaving 80 participants in the final analysis. This rejection criterion, as well as the experimental procedures and planned analyses described below, were preregistered through AsPredicted.^7^

##### Materials.

This experiment employed a 4 × 2 within-participants design, crossing scene type (4 levels) and motion verb (perspectival versus non-perspectival).

**Figure 7.**
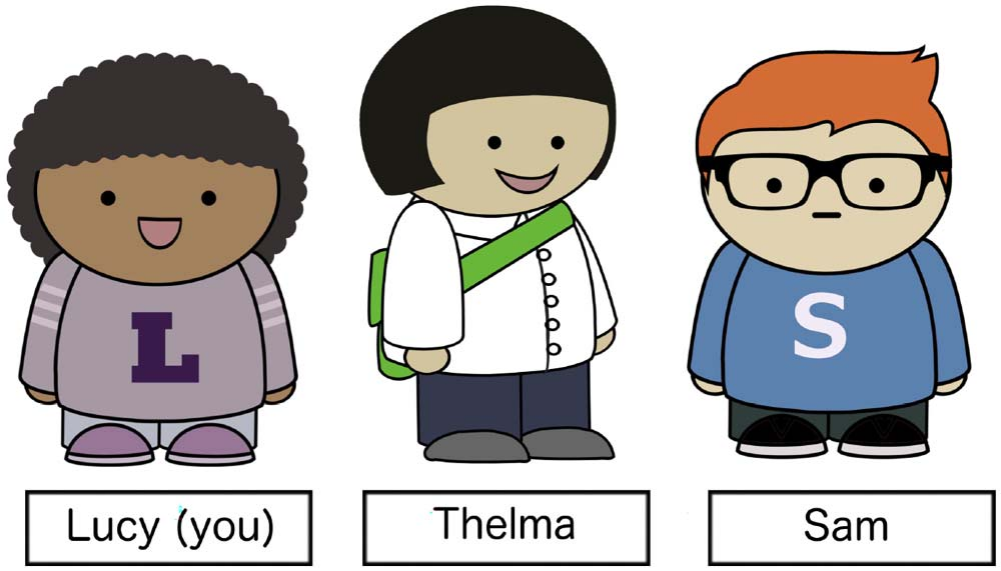
Stimuli characters.

The materials used three cartoon characters: Sam, Lucy, and Thelma (Figure 7). In the comprehension experiment, participants were asked to imagine themselves as Lucy. Participants were introduced to Sam, Lucy’s friend, and told that Sam “sometimes gets confused and says things that don’t make sense.” Their goal was, given an illustration of a scene, to decide whether what Sam was saying was appropriate in the context.

There were 4 versions of the scene: one with both the speaker and listener at the destination of motion (Both scene); one with the speaker at the destination and the listener at the other location (Speaker scene); one with the listener at the destination and the speaker at the other location (Listener scene); and one where neither the listener nor the speaker were at the destination (None scene). An example of a Both scene is shown in Table 8 (left). In scenes in which the speaker and listener are not located in the same place, they were depicted talking to each other on the phone (Table 8 right).

**Figure 8.**
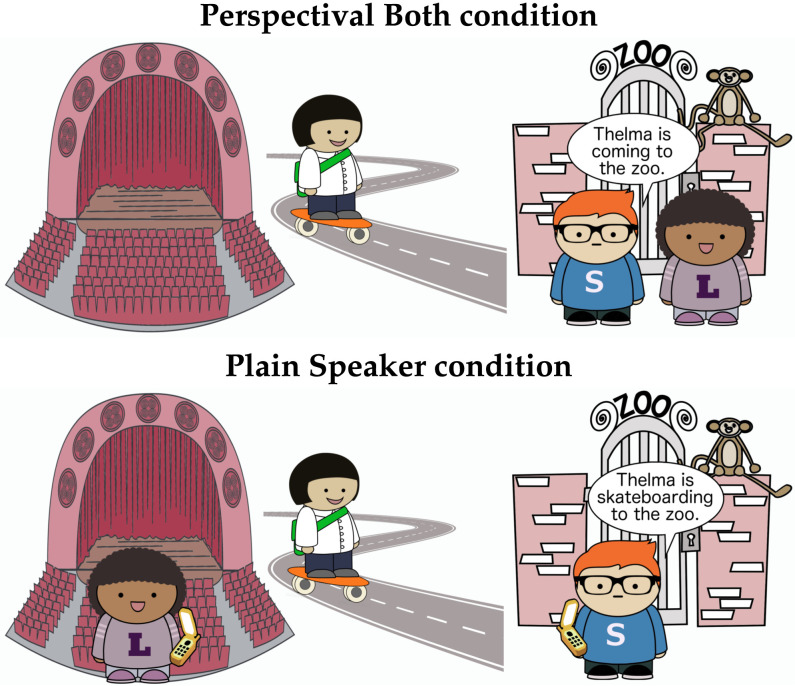
Comprehension stimuli.

Each scene was matched with two versions of the sentence: one using *come*, the perspectival condition; and one using a manner-of-motion verb such as *walk* or *drive*.^8^ Thus, there were two sentence conditions: Perspectival and Plain. The Plain conditions were introduced as a control condition to index the baseline difficulty of processing the different scenes. We had no a priori predictions for RT rates for the Plain conditions. We reasoned that any processing boost for a scene that matched the perspective assigned to a perspectival motion verb should occur above and beyond the ease of processing the scene.

##### Spatial Control Task.

Our experiment rests on the ability of participants to adopt the perspective of the listener character. If they are unable to fully take this character’s perspective, the external validity of our task is suspect. To measure how well participants adopted Lucy’s perspective, we included spatial control items throughout the experiment. These items set up contrasts between the participant’s visual perspective and Lucy’s perspective.

We included three kinds of contrasts. In the Close condition of the spatial control items, Sam makes a statement about the item closest to him in the scene. The scene depicts an object close to Sam and a distractor object that is closest to the scene viewer (the participant’s visual perspective). In the Between condition, Sam makes a statement about an object between Lucy and a reference point, or between himself and a reference point. There is a distractor between the reference point and scene viewer. The third condition involved *right* and *left* contrasts between Sam and the participant’s visual perspective. This condition was predicted to be more challenging and was not used to exclude participants.^9^

**Figure 9.**
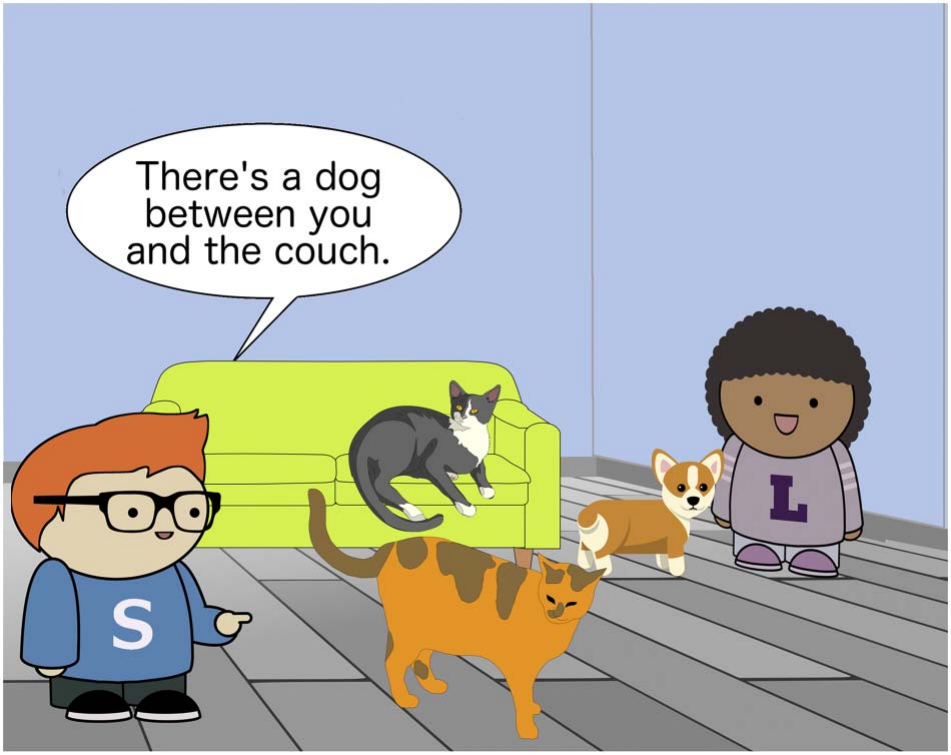
Comprehension spatial stimulus.

An example spatial control item is shown in Figure 9. In this scene, the animal between the participant and the couch is a cat, but the animal between Lucy and the couch is a dog. This description-scene pair should be accepted only if participants have adopted Lucy’s spatial perspective.

Participants who missed more than 1 item from the Between and Close conditions were excluded from the study. We rely on this measure to determine whether participants successfully adopted Lucy’s perspective as instructed.

##### Filler Items.

**Figure 10.**
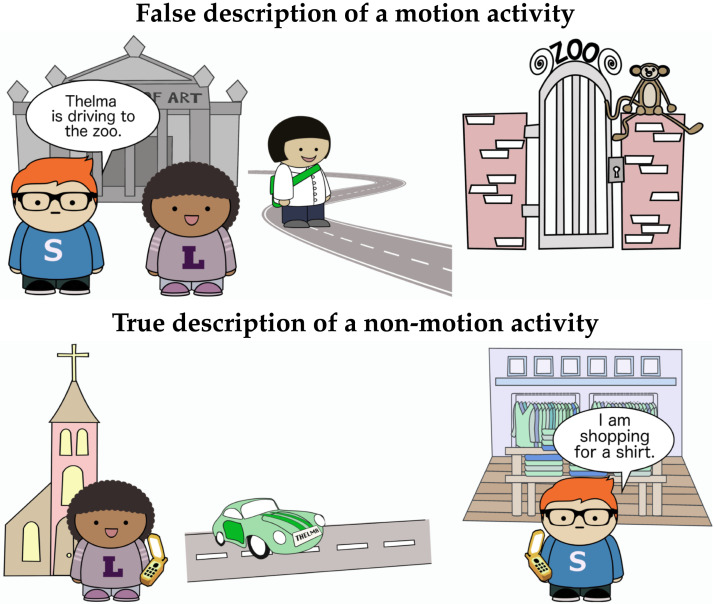
Comprehension stimuli.

In addition to the 20 spatial control items discussed above, 30 filler items were included. Like the main items, these items depicted the three characters Thelma, Sam, and Lucy in scenes with two possible locations. Most filler items depicted non-motion activities. Some showed Thelma moving, but towards a location on the left rather than the right. Others showed the speaker or listener characters moving. 20 filler items were paired with false descriptions, and 10 were paired with true descriptions (Figure 10).

##### Procedure.

There were 4 (scene type) × 2 (motion verb) conditions. Participants saw 6 items in each of the 8 conditions. Items were distributed into 8 Latin Square lists, and combined with 30 fillers and 20 spatial control items, for a total of 98 items. Participants also completed 4 training items before the experiment began. Stimuli were displayed and responses collected using the Ibex Farm platform for web-based experiments (Drummond, 2019). Each experimental session began with an informed consent form and concluded with a demographic survey and a debriefing survey, which allowed participants to report any issues with the survey and contained two free-response bot-check questions.

For each experimental item, participants were first shown an image of Sam’s head with a speech bubble containing the target sentence. Then they saw a scene depicting the conversation, and were given 10 seconds to indicate whether or not the picture and sentence matched. If they did not respond within this time window, they were told that they were too slow, and the experiment moved to the next item.

Both reaction times (how quickly, in milliseconds, participants responded) and acceptance rate (how often participants indicated that the scene and sentence were compatible) were measured.

#### Analysis.

Two mixed-effects regression models were fitted to the data: a linear model for the reaction time data, and a logistic model for the acceptance rate data. The reaction time data analysis was limited to trials in which participants accepted the scene/sentence pair.

The maximal random effects structure was used for both models: random intercepts and slopes were included for all fixed-effects predictors, for participants, and for items. The models were fitted using the lme4 package in R (Bates et al., 2015). Treatment coding was used in both models, with the Plain Speaker condition as the baseline. This resulted in the following fixed-effects contrasts: Perspectival, 1 for items in the Perspectival condition and 0 for items in the Plain condition; Both, 1 for the Both condition and 0 otherwise; Listener, 1 for the Listener condition and 0 otherwise; and None, 1 for the None condition and 0 otherwise.

Of critical importance for testing the predictions of the models under consideration is how quickly participants can accept a perspectival verb as a valid description of the different scenes relative to that scene’s Plain baseline. Treating the Speaker condition as the baseline allows us to interpret the Perspective:Both interaction term as measuring this key comparison of interest, since it takes into account the differences between reaction times for scene types in the Plain versus the Perspectival condition. We therefore look at differences between each participant’s average reaction times for the Plain condition and their average reaction times for the Perspectival condition for each scene type.

#### Results.

**Figure 11.**
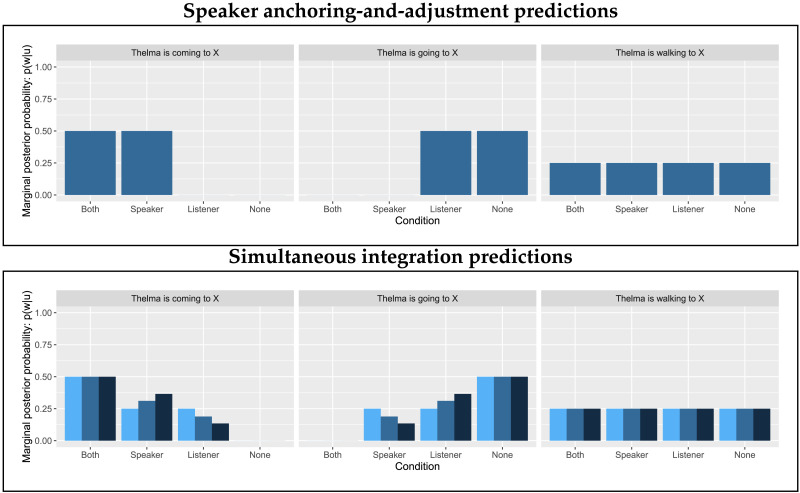
Speaker anchoring-and-adjustment and simultaneous integration model predictions for comprehension.

The predicted probabilities of each scene by sentence according to the speaker anchoring-and-adjustment and simultaneous integration models are shown in Figure 11. Critically, the simultaneous integration model predicts highest probability for the Both scene when the sentence uses *come*, while the speaker anchoring-and-adjustment model predicts that the Speaker and Both scenes will be equally likely.

##### Reaction Time Results.

**Figure 12.**
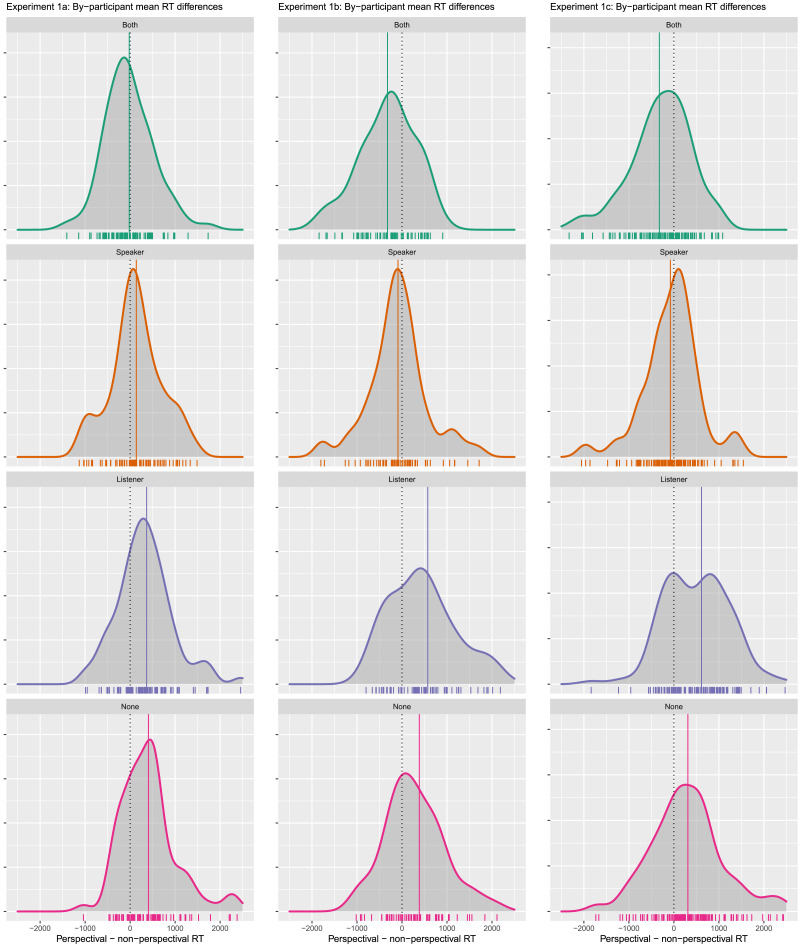
**Experiments 1a–1c distribution of by-participant differences in RTs to non-perspectival verbs minus RTs to perspectival verbs.** Negative values indicate that perspectival verbs were accepted faster than their non-perspectival counterparts for a given scene; positive values indicate that perspectival verbs were accepted more slowly than their non-perspectival counterparts. Hatch marks indicate individual values; the vertical line indicates the distribution mean.

Figure 12 shows the distribution of by-participant differences in reaction times to each type of scene for Plain and Perspectival verbs. To calculate this, a participant’s Perspectival RT was subtracted from their Plain RT by scene. Negative values of this difference indicate that acceptance times for the Perspectival condition were faster than for its Plain counterpart.

Reaction times in the Perspectival Both condition were faster than in the Plain Both condition. In all other conditions, reaction times were slower in the Perspectival condition than the Plain condition. A reliable difference in reaction times for the Both and Speaker scenes is supported by the mixed-effects model shown in Table 1, which finds a significant interaction between Both and Perspectival.

**Table 1.** Experiment 1a RT mixed effects regression analysis, fixed effects (*n* = 3630)

**Fixed effects**	$\hat{\beta }$	***z***	***p***
Perspectival	0.06(± 0.02)	3.16	< **0.01**

Both	0.03(± 0.02)	1.43	0.152
Listener	−0.03(± 0.02)	−1.28	0.201
None	−0.08(± 0.02)	−4.14	< **0.0001**

Perspectival:Both	−0.06(± 0.03)	−2.22	< **0.05**
Perspectival:Listener	0.03(± 0.03)	1.05	0.295
Perspectival:None	0.05(± 0.03)	1.65	0.101

##### Acceptance Rate Results.

**Figure 13.**
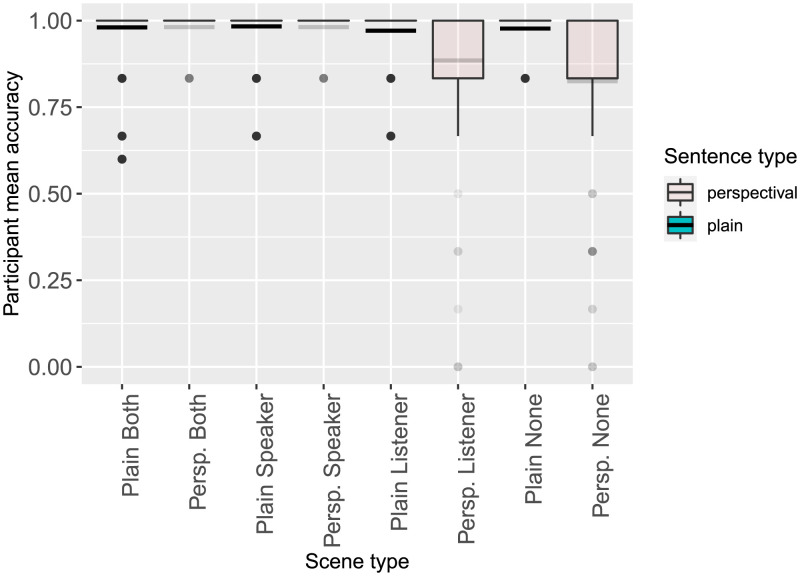
Experiment 1a participant mean acceptance rates by condition.

The acceptance rate results (Figure 13) were at ceiling in most conditions, including in the critical conditions (Perspectival Speaker and Perspectival Both). This means that the acceptance data cannot be interpreted in support of either model. Surprisingly, the acceptance rates for the Perspectival None condition were also quite high: items in this condition were accepted 83% of the time.

#### Discussion.

The results of Experiment 1a provide qualified support for the existence of a Convergent Perspective Boost, as predicted by the simultaneous integration model. Participants were faster to accept scene-sentence pairs in the Perspectival Both condition than in the Perspectival Speaker condition, supporting the idea that listeners reason simultaneously over multiple perspectives. However, no evidence of a Convergent Perspective Boost was observed in the acceptance rate data. In both the Perspectival Speaker and Perspectival Both conditions, participants’ acceptance rates were at ceiling (as they were in all Plain conditions).

The rate of acceptance of *come* in the None scenario was troublingly high, given that the scene does not satisfy the perspectival requirement in the semantics of *come*. The high acceptance rates overall may indicate an experimental design issue. Responses to the debriefing questions suggest that some participants may have hesitated about rejecting items where the scene was valid for the truth-conditions of the sentence, but not the perspectival anchoring of *come*. In addition, the experimental design may have biased participants towards positive responses, given that 7 of the 8 conditions were predicted to be accepted, leading to inflated acceptance rates across the board.

To address this limitation, we ran two replication studies with small modifications.

### Experiments 1b and 1c

Experiments 1b and 1c are sentence/picture compatibility judgment tasks replicating Experiment 1a. In Experiment 1b, additional fillers were introduced to encourage participants to reject items. Participants were also given more explicit instructions about when to reject items. Experiment 1c employs the same paradigm as Experiment 1b, but with a larger sample size. In addition, in Experiment 1c, we recruited two balanced groups of participants by race to investigate the possibility that participants may be better able to access the Listener perspective if the perceived race of the Listener character matches their own.

#### Methods

##### Participants.

For Experiment 1b, 95 monolingual American English-speaking participants were recruited through Prolific. Participants who achieved less than 90% accuracy on the spatial control task were excluded from the experiment (*n* = 31). This left a total of 64 participants in the final analysis.

For Experiment 1c, 223 participants were recruited in two groups. One group consisted of monolingual American-English speaking participants who identified as Black, recruited through Prolific. The other consisted of monolingual American-English speaking participants who identified as white, also recruited through Prolific. Participants who achieved less than 90% accuracy on the spatial control task were excluded from the experiment (*n* = 95), leaving 128 participants divided evenly across the two groups. This rejection criterion, as well as the experimental procedures and planned analyses described below, were preregistered through the Open Science Foundation.^10^

##### Materials.

In both replications, the critical stimuli and fillers from Experiment 1a were used.

**Figure 14.**
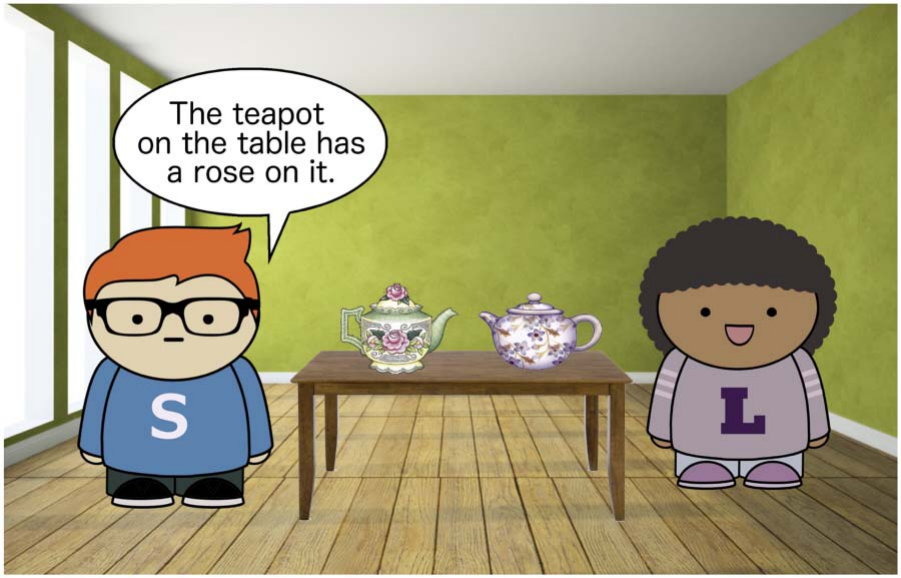
Pragmatically odd filler example: definiteness violation.

To address the positive response bias in Experiment 1a, both replications used additional filler items that were designed to be grammatical, but pragmatically bad. 15 fillers were added: 5 definiteness violations, 3 presupposition violations, 3 scalar implicature cases, and 4 under-specific number cases. Figure 14 shows an example definiteness violation filler: using *the teapot on the table* in this context violates the definiteness presupposition of *the* because there are two teapots on the table. The total number of items was therefore increased to 113.

##### Procedure.

The procedure was largely identical to Experiment 1a. However, the instructions to participants were modified in order to encourage them to reject pragmatically odd, but truth-conditionally valid descriptions. Participants were instructed that Sam “sometimes says things in a weird way or says things that don’t make sense,” and they were told that their task was to indicate whether or not what Sam says seemed “normal” according to the picture. This was meant to prompt them to reject uses of come without a valid perspectival anchoring.

Participants were also given more training with pragmatically odd items, and more feedback about why those items should be rejected. They were shown 6 training items: 2 pragmatic violations, 1 false description, and 3 valid descriptions, including a normal description of an odd scene, to help them differentiate between unusual scenarios and unusual descriptions.

#### Analysis.

Mixed-effects models were fit to the reaction time data as described for Experiment 1a.

#### Results

##### Reaction Time Results.

In both replications, mean reaction times were fastest in the Perspectival Both condition, as in Experiment 1a (Figure 12).

In Experiment 1b, the critical interaction between the Perspectival and Both conditions was not significant (Table 2). In Experiment 1c, as in Experiment 1a, the interaction was significant (Table 3).

**Table 2.** Experiment 1b RT mixed effects regression analysis, fixed effects (*n* = 2581)

**Fixed effects**	$\hat{\beta }$	***z***	***p***
Perspectival	−0.03(±0.03)	−1.1	0.28

Both	0.005(±0.02)	0.2	0.85
Listener	−0.037(±0.03)	−1.4	0.15
None	−0.12(±0.03)	−5.1	< **0.0001**

Perspectival:Both	−0.06(±0.03)	−1.9	0.059
Perspectival:Listener	0.19(±0.04)	5.2	< **0.0001**
Perspectival:None	0.13(±0.05)	2.6	**0.01**

**Table 3.** Experiment 1c RT mixed effects regression analysis, fixed effects (*n* = 3103)

**Fixed effects**	$\hat{\beta }$	***z***	***p***
Perspectival	−0.01(±0.02)	−0.6	0.53

Both	0.03(±0.02)	1.8	0.08
Listener	−0.004(±0.02)	−1.2	0.82
None	−0.063(±0.02)	−3.3	**0.001**

Perspectival:Both	−0.087(±0.02)	−3.5	< **0.001**
Perspectival:Listener	0.16(±0.03)	5.4	< **0.0001**
Perspectival:None	0.08(±0.03)	2.5	**0.02**

##### Acceptance Rate Results.

**Figure 15.**
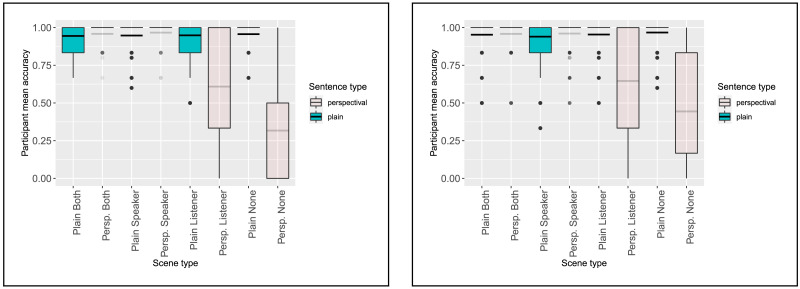
Experiment 1b (left) and 1c (right) participant mean acceptance rates by condition.

The addition of the pragmatically odd fillers was effective at decreasing the acceptance rate for Perspectival None items (Figure 15). However, in both replications, the acceptance rates for the Speaker and Both conditions remained at ceiling. Mixed-effects logistic regression models found no reliable differences between the rates of acceptance in the Perspectival Both and Perspectival Speaker conditions for either experiment (Tables 4 and 5).

**Table 4.** Experiment 1b acceptance mixed effects regression analysis, fixed effects (*n* = 2581)

**Fixed effects**	$\hat{\beta }$	***z***	***p***
Perspectival	0.71(±0.4)	1.7	0.09

Both	1.1(±0.6)	1.7	0.08
Listener	−0.02(±0.3)	−0.06	0.96
None	0.64(±0.4)	1.5	0.1

Perspectival:Both	0.09(±0.7)	0.13	0.9
Perspectival:Listener	−3.5(±0.5)	−7.4	< **0.0001**
Perspectival:None	−6.0(±0.6)	−10.7	< **0.0001**

**Table 5.** Experiment 1c acceptance mixed effects regression analysis, fixed effects (*n* = 3103)

**Fixed effects**	$\hat{\beta }$	***z***	***p***
Perspectival	0.9(±0.3)	3.0	**0.002**

Both	0.8(±0.3)	2.7	**0.007**
Listener	0.4(±0.3)	1.7	0.09
None	0.7(±0.3)	2.5	**0.01**

Perspectival:Both	0.5(±0.5)	0.96	0.34
Perspectival:Listener	−3.1(±0.5)	−7.7	< **0.0001**
Perspectival:None	−5.2(±0.5)	−10.98	< **0.0001**

In both replications, the rate of acceptance of Perspectival Listener items decreased substantially. Although both models predict that listeners should expect the speaker to be located at the destination of motion (possibly in addition to the listener), each also predicts that listener-oriented readings should be available when the speaker’s perspective is ruled out by context, as in the Perspectival Listener condition. So although both models predict slower reaction times in this condition (for different reasons), the low rate of acceptability in this condition is surprising.

### Combined Discussion

When considered together, 1a–1c provide support for the simultaneous integration account’s prediction that listeners take into account multiple perspectives at once when interpreting grammatically perspectival expressions. In all three comprehension experiments, we saw that the reaction times were numerically fastest in the Perspectival Both condition; in two out of three experiments RTs were significantly faster in the Perspectival Both condition than the Perspectival Speaker condition. This pattern suggests a Convergent Perspective Boost, as predicted by the simultaneous integration view. In contrast, the data do not seem to support the Simple Speaker Advantage predicted by the speaker anchoring-and-adjustment model.

There are two qualifications necessary to this conclusion. One is that the predicted Convergent Perspective Boost was not observed in the acceptability measure: acceptance rates were at ceiling in both conditions. The evidence for the Convergent Perspective Boost comes entirely from the RT data: participants were fastest to accept the scene where both potential perspective-holders were at the destination following *Thelma is coming*. The second is that we did not observe a significant interaction in Experiment 1b. Given the relatively small sample size of this experiment compared to the others, we believe that this is most likely due to low power.

Finally, in Experiment 1c, two groups of participant were recruited in order to explore whether participants find it easier to adopt the listener character’s perspective when they share more demographic characteristics, since the experimental stimuli depicted a Black listener character. An exploratory meta-analysis of the acceptance rates in Experiment 1a and 1b by race suggested that Black participants considered the listener’s perspective more often: in a post-hoc mixed-effects regression analysis, their Listener Perspectival acceptance rates were reliably higher than other participants. However, though acceptance rates in the Perspectival Listener condition were higher among Black participants, a post-hoc mixed-effects regression analysis found no reliable between-group differences in either acceptance rates or reaction times in Experiment 1c. Although we did not find a reliable effect of race, we feel that the effect of shared demographic characteristics on grammatical perspective-taking is an interesting direction for future work.

## PERSPECTIVE SELECTION IN PRODUCTION

In production, the speaker must select a compatible perspective and utterance to communicate their intended meaning. In this section, we illustrate two key differences in the production predictions of the speaker anchoring-and-adjustment and simultaneous integration models of grammatical perspective-taking.

### Speaker Anchoring-and-Adjustment Production Predictions

The production version of the speaker anchoring-and-adjustment posits that speakers always use their own perspectives. Unlike in comprehension, there is no cue to prompt the selection of a different perspective, since there is always an utterance compatible with the intended meaning and the speaker’s perspective. If the speaker is at the destination of motion, they can use *come*; if they are not, they can simply choose a different verb, like *walk* or *go*. The production task is therefore less constrained than comprehension, since the speaker can choose from a wide space of possible utterances.

Consequently, the speaker anchoring-and-adjustment production model predicts that speakers will always use their own perspectives. They will produce *come* only when describing scenes where they are located at the destination of motion (Both, Speaker). This gives rise to the same Simple Speaker Advantage outlined in comprehension: *come* is most likely to be used to describe scenes where the speaker is at the destination. For scenes where the speaker is not at the destination of motion, speakers are expected to produce *go* or *walk* rather than *come* (Listener, Speaker Moving Listener, None, Speaker Moving None).

The speaker anchoring-and-adjustment model does not predict any difference in the speaker’s behavior when describing scenes where Thelma is the mover compared to scenes where the speaker is the mover.^11^ In both cases, they will use *go* or *walk* to describe scenes where they are not located at the destination of motion rather than shifting to a perspective that licenses *come*. So, the speaker anchoring-and-adjustment model predicts that the speaker will describe all scenes where they are the mover with *go* or *walk*, since the speaker cannot both be in motion and located at the destination of motion simultaneously.

### Simultaneous Integration Production Predictions

In the simultaneous integration model of production, speakers consider multiple perspectives at once. They reason about the listener’s comprehension process in order to select the utterance-perspective pair that best describes their intended meaning.

Because speakers consider multiple perspectives simultaneously, the utility of *come* is predicted to be highest when it describes a scene where all potential perspective-holders are at the destination of motion. In this case, *come* is licensed for all perspectives that the listener might take. If the speaker uses *come* to describe a scene where only one perspective-holder is at the destination, they run the risk that the listener might select the wrong perspective, and consequently, mis-interpret their utterance.

As a result, the simultaneous integration model predicts a Convergent Perspective Boost: speakers should produce *come* most often when describing the Both scene. Speakers are also predicted to produce *come* at a less frequent rate in the Speaker and Listener scenes; the relative frequency will vary with the strength of the penalty for non-speaker perspectives. The speaker is not predicted to produce *come* in the None scene.

So far, these predictions parallel those of the comprehension model. However, in production, the predictions of the simultaneous integration and speaker anchoring-and-adjustment models differ in a second respect. The simultaneous integration model predicts a benefit from decreased ambiguity when the speaker is the mover.

In the Speaker Moving Listener scene, the speaker is the mover, and therefore, cannot serve as the perspective-holder for *come*. This means that the ambiguity over perspective-holders for *come* is eliminated in this scene: the perspective-holder must be the listener. Because the simultaneous integration model incorporates reasoning over the listener’s interpretation process, it consequently assigns high utility to *come* as a description of the Speaker Moving Listener scene compared to the Listener scene.

We refer to this as the **Ambiguity Elimination Advantage**: the simultaneous integration model predicts more frequent use of *come* in scenes where there are fewer potential perspective-holders for listeners to consider. By contrast, the speaker anchoring-and-adjustment model predicts no difference between the Listener and Speaker Moving Listener scenes: in both cases, *come* is not predicted to be produced, since it would require adopting a non-speaker perspective.

### Summary

**Figure 16.**
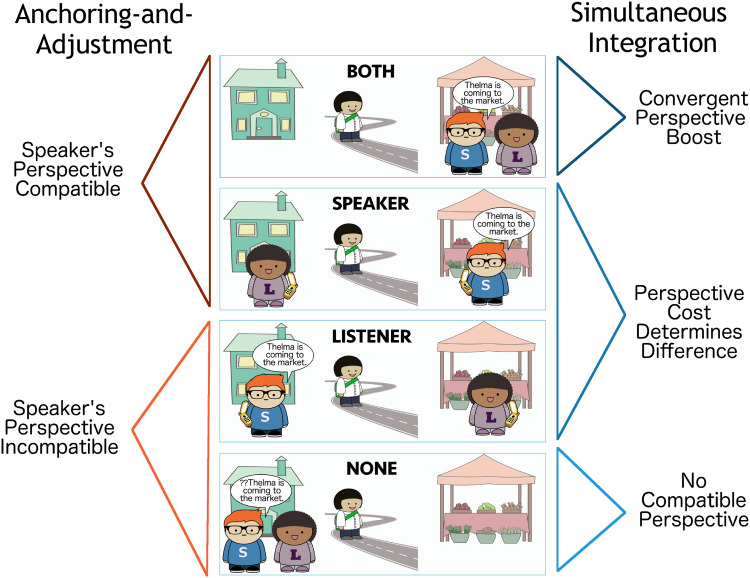
Ranked likelihood of uttering *come* in each scene as ranked by Convergent Perspective Boost versus Simple Speaker Advantage.

In production, the predictions of the speaker anchoring-and-adjustment and simultaneous integration models differ in two key ways. First, as in comprehension, the simultaneous integration production model predicts a Convergent Perspective Boost (*come* will be produced most when describing the Both scene), while the speaker anchoring-and-adjustment model predicts a Simple Speaker Advantage (*come* will be produced equally often when describing the Speaker and Both scenes). The most likely contexts for speakers to produce *Thelma is coming* when Thelma is the character in motion under each model are depicted in Figure 16.

One interpretative difficulty is that if the cost of non-speaker perspectives is high, the Convergent Perspective Boost may be too small to be observed. If the distribution over perspectives is highly skewed towards the speaker or the cost for non-speaker perspectives is sufficiently high, the listener’s perspective will be sampled so infrequently that the perspective set will appear to contain only the speaker’s perspective. Consequently, if *come* is used more in the Both condition, the evidence supports the simultaneous integration model, but if *come* is used equally in the two conditions, the evidence is compatible with either a speaker anchoring-and-adjustment model or a simultaneous integration model with a high perspective cost.

**Figure 17.**
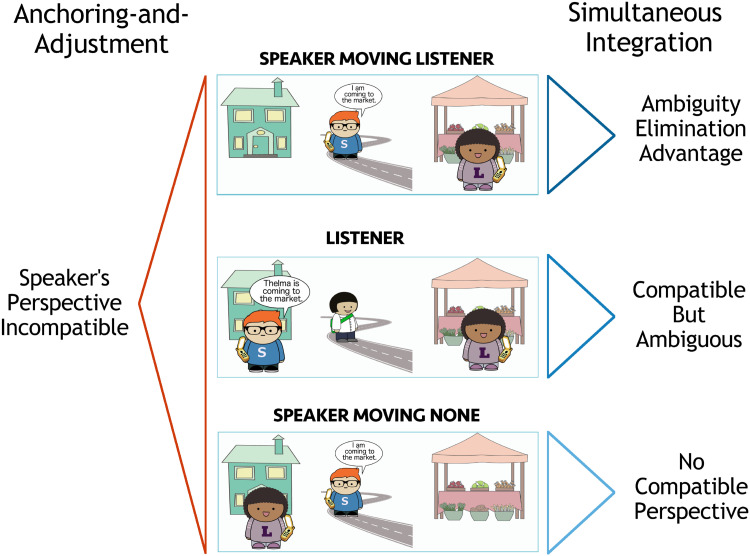
Ranked likelihood of uttering *come* in each scene as ranked by Ambiguity Elimination Advantage versus Simple Speaker Advantage.

However, the predictions of the two models also differ for a second scenario: when the speaker is the character in motion. The simultaneous integration model predicts a difference between the rates of *come* production in the Listener and Speaker Moving Listener scenes: under the Ambiguity Elimination Advantage, *come* should be produced more often in the latter condition. The speaker anchoring-and-adjustment model predicts that *come* should not be used to describe either scene, since this would mean adopting a non-speaker perspective. The predicted likelihood of speakers producing an utterance containing *come* according to each model in the Listener condition and two Speaker Moving conditions are depicted in Figure 17. The key difference between the model predictions for contexts in which the speaker is the mover does not disappear when the cost of non-speaker perspectives is very high in the simultaneous integration model, since there should still be an observable Ambiguity Elimination Advantage in the Speaker Moving Listener condition.

## PRODUCTION EXPERIMENTS

In this section, we describe the results of two production experiments that compare the predictions of the speaker anchoring-and-adjustment and simultaneous integration models of grammatical perspective-taking. In production, the speaker’s task is to select the utterance and perspective that best communicate their intended meaning.

As in the comprehension experiments, we use illustrated scenes to depict observed worlds (meanings). In both experiments, participants were shown a scene and asked to complete an utterance naturally in the given context. We interpret the rate at which participants produce *come*, *go*, and manner-of-motion verbs in each condition as a measure of the marginal posterior probability of the utterance given the depicted world.

### Experiment 2a

Experiment 2a is a speech bubble completion task that tests the existence of a Convergent Perspective Boost in production. Critically, we asked whether there were more productions of *come* for Both scenes than for Speaker scenes, as predicted by the simultaneous integration model, or whether speakers produced *come* at a similar rate for Both and Speaker scenes, as predicted by the speaker anchoring-and-adjustment model. The experimental procedures and planned analyses described below were preregistered through the Open Science Foundation.^12^

#### Methods

##### Participants.

42 Monolingual American English-speaking participants were recruited through Prolific. One participant was excluded after giving incoherent answers to bot-check questions, and another was excluded because they reported mixing up the speaker and listener characters. This exclusion was not preregistered, as it was unforeseen.

##### Materials.

Experiment 2a employed a 4 × 1 within-participants design with the same 4 scene types from the comprehension experiments: Both, Speaker, Listener, and None.

**Figure 18.**
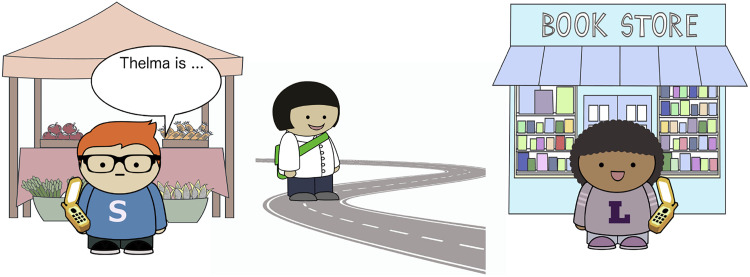
Experiment 2a example stimulus in the Listener condition.

The scene stimuli were modified slightly for the production experiments. In the production paradigm, the speech bubble in each scene showed only the beginning of a sentence (*Thelma is* …), which participants were asked to complete. To encourage participants to focus on the motion event, the scenes were edited to show the same manner of motion (walking). Figure 18 shows an example Listener scene.

The filler and spatial control items from Experiment 1a were used as fillers for both production experiments, modified in the same way as the main condition items. Unlike in the comprehension experiments, the spatial control items were not used to exclude participants, due to the difficulty of prompting them to describe the critical contrast between the speaker’s visual perspective and their own.

##### Procedure.

In the production study, participants were asked to imagine themselves as the speaker character, Sam. Unlike in the comprehension studies, where participants saw the sentence before the scene, in the production study, participants saw the scene with a partially completed sentence within the speech bubble (Figure 18). Participants were asked to type a completion for the sentence in a text box. They were told to complete the speaker’s sentence as naturally as possible, and to only mention what was shown in the scene. In the main conditions, the sentence prompt was “Thelma is ….” In the filler and spatial control items, the prompt varied based on the scene.

Participants saw 12 items in each of the 4 scene conditions (Both, Speaker, Listener, None). Stimuli were displayed and responses collected using the Ibex Farm platform for web-based experiments (Drummond, 2019). Each experimental session began with an informed consent form and concluded with a demographic survey and a debriefing survey, which allowed participants to report any issues with the survey and contained two free-response bot-check questions.

#### Data Coding.

**Figure 19.**
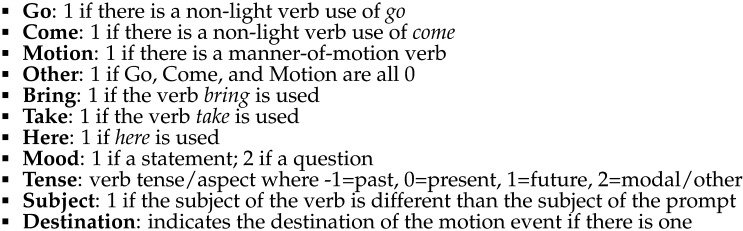
Experiment 2a data coding categories.

Participant responses were coded for 11 categories, as shown in Figure 19. Categories were not exclusive: a response might contain both an instance of *come* and another verb. There were two annotators: one of the authors and an annotator who was unaware of the purpose of the experiment. The inter-annotator agreement scores for each category ranged from 0.89–1 (Cohen’s *κ*).

The primary dependent variables of interest are the rates of *come*, *go*, and manner-of-motion verbs. The tense, mood, subject, and destination categories were used to identify in-progress motion descriptions. Responses that were questions or did not describe an ongoing event were excluded.^13^ For instance, the response shown in (7) was excluded: although it contains a motion verb, it is within a question.

7.  *Thelma is* … coming to the pet store? Nice.

Across both production experiments, around 11% of responses were excluded because they did not describe an ongoing event. This rate was roughly constant across conditions. Motion descriptions with destinations or subjects other than the one depicted in the scene were also excluded; these were assumed to result from misinterpretations of the scene. Around 20% of responses were excluded because they mentioned the wrong destination or no destination. This exclusion rate was roughly equal across conditions, though lower in the None condition (10%). No responses mentioned the wrong subject.

The *go* category includes only uses of *go* as a motion verb. Across Experiments 2a and 2b, 33 out of the 1448 responses containing a lemma of *go* were excluded because they did not meet this criteria. About half of these were in the phrase *going shopping*. Most of the rest were future auxiliary uses of *go*.^14^

#### Analysis.

The rate of *come* responses was analyzed using a mixed effects logistic regression model. The model used four fixed-effects predictors, corresponding to the four scenes: Both, Speaker, Listener, None. The maximal random effects structure was used: random intercepts and slopes were included for all fixed-effects predictors, for participants, and for items. The model was fit to the coded data using the lme4 package in R (Bates et al., 2015), and all pairwise comparisons were extracted from the resulting model using the multcomp package (Hothorn et al., 2008).^15^

#### Results.

**Figure 20.**
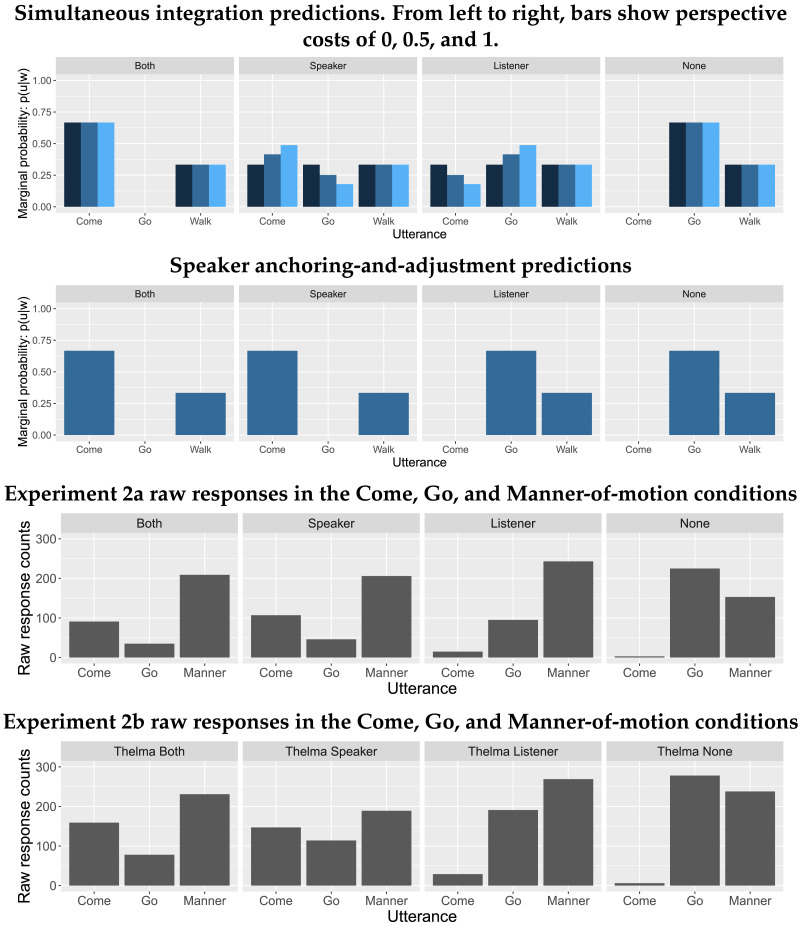
Main condition production predictions and results.

Figure 20 displays the raw responses for the three main categories of interest along with the predictions generated by the speaker anchoring-and-adjustment and simultaneous integration models.

The simultaneous integration model predicts that *come* will be produced most frequently in the Both condition, followed by the Speaker condition. It predicts the opposite pattern for *go*: it is most likely in the None condition, followed by the Listener condition, and then the Speaker condition (based on the strength of the speaker bias). The speaker anchoring-and-adjustment model predicts that *come* will be produced equally often in the Speaker and Both conditions. For *go*, it predicts equally frequent use in the Listener and None conditions, and no use in the Both and Speaker conditions.

We note that we expect the rates of production for *come* and *go* to be lower than predicted by the model, since our model considers only one manner-of-motion verb, while production participants were unconstrained in their choice of motion description.

Table 6 shows the proportion of *come*, *go*, and manner-of-motion verbs used in participant responses that described the motion event (1428 of the original 1872 responses).

**Table 6.** Experiment 2a proportion of motion responses by condition and type, along with raw counts

**Condition**	***come***	***go***	***walk* etc**	***n* motion responses**
Both	27%	10%	63%	335
Speaker	30%	13%	58%	359
Listener	4%	27%	69%	353
None	0.8%	59%	40%	381

Participants used *come* most frequently to describe the Speaker scene, followed by the Both scene. A high rate of manner-of-motion completions was found across conditions, with the highest rate in the Listener condition (71%). The proportion of Listener completions using *come* was very low (4%).

The None condition was the only one in which manner-of-motion completions were not used more frequently than either perspectival verb. In the None condition, *go* was strongly preferred, as both models predict. The proportion of None condition responses involving *come* was very small, as expected.

The pattern of *go* use roughly follows the predictions of the Simultaneous Integration model: it is highest in the None condition, followed by the Listener, and then the Speaker conditions. Contrary to the predictions of both models, however, it was used sometimes in the Both condition.

Although the proportion of *come* responses was numerically higher in the Speaker condition than the Both condition, this difference was not reliable in the mixed-effects model (Table 7).

**Table 7.** Experiment 2a *come* response mixed effects logistic regression analysis

**Fixed effects (*n* = 1872)**	$\hat{\beta }$	***z***	***p***
(Intercept)	−1.66(±0.26)	−6.4	< **0.0001**
Both	−0.37(±0.26)	−1.5	0.15
Listener	−4.03(±0.6)	−6.7	< **0.0001**
None	−15.5(±4.1)	−3.8	**0.0002**

Despite the low rate of *come* responses in the Listener condition, the mixed effects model found a reliable difference in the rate of *come* responses in the None and Listener conditions (Table 8).

**Table 8.** Pairwise comparisons between Scene conditions in mixed effects logistic regression analysis

**Linear hypothesis**	$\hat{\beta }$	***z***	***p***
Both-Speaker==0	−0.37(±0.3)	−1.5	0.4
Listener-Speaker==0	−4.03(±0.6)	−6.7	< **0.001**
None-Speaker==0	−15.48(±4.1)	−3.8	< **0.001**
Listener-Both==0	−3.65(±0.6)	−5.7	< **0.001**
None-Both==0	−15.11(±4.1)	−3.7	< **0.001**
None-Listener==0	−11.45(±4.1)	−2.8	**0.0178**

#### Post-hoc Spatial Controls Analysis.

A main concern about the validity of our experiment is that participants may not be successfully adopting the perspective of the character that they are instructed to take on. In the comprehension experiments, we use the spatial control task to exclude participants who did not demonstrate the ability to adopt Lucy’s perspective. In the production experiments, we did not use the spatial controls as an exclusion mechanism, because participants had freedom over what part of the scene to describe; many participants did not describe the aspect with the critical contrast between the speaker character’s visual perspective and their own.

To explore whether the ability of participants to take on the speaker’s perspective poses a threat to the external validity of our task, a post-hoc analysis of the spatial control items was performed, as described in Appendix A. For the Between and Close conditions, 89% of the responses were true according to the speaker’s perspective and not the visual perspective of the participant. 0.5% were true according the visual perspective of the participant and not the speaker character. This suggests that in the majority of trials, participants were adopting the speaker character’s perspective rather than relying on their own.

#### Discussion.

The main finding of interest in Experiment 2a is that speakers did not produce come more often when describing the Both scene than the Speaker scene; in fact, a numerical trend was observed in the opposite direction. Unlike the comprehension experiments, this experiment found no evidence of a Convergent Perspective Boost ; instead, the data are more consistent with a Simple Speaker Advantage. Although the raw percentages of *come* and *go* in the production data are not expected to match up with the comprehension measures, since the production task is more open-ended, this is evidence of an asymmetry between the comprehension results and the production results.

We note, however, that two facts may complicate the interpretation of the data. The lack of a Convergent Perspective Boost and a low rate of *come* responses in the Listener condition together could suggest a strong egocentric bias. A strong bias towards sepaker perspectives complicates the interpretation of the lack of a Convergent Perspective Boost, since as the cost of non-speaker perspectives increases in the simultaneous integration model, the predicted Convergent Perspective Boost decreases. It is also possible that our sample size was too small to observe a Convergent Perspective Boost. Unlike in the comprehension task, participants were not constrained to consider only motion verbs, so the observed rate of responses that include perspectival motion verbs was fairly low in all conditions.

### Experiment 2b

Experiment 2b is a replication and extension of Experiment 2a. It tests two predicted differences between the speaker anchoring-and-adjustment and simultaneous integration models of production: the existence of a Convergent Perspective Boost, and the existence of a Ambiguity Elimination Advantage.

Experiment 2a found no evidence of a Convergent Perspective Boost. However, as noted above, as the strength of egocentric bias increases, the Convergent Perspective Boost is predicted to decrease. Therefore, in this experiment, we also test a second difference between the predictions of the simultaneous integration and speaker anchoring-and-adjustment models: whether a reduction in perspective-holder ambiguity affects how frequently speakers use *come*.

#### Methods

##### Participants.

63 monolingual American English-speaking participants were recruited through Prolific. 7 participants were excluded because they gave incoherent answers to the bot-check questions, leaving 56 participants. This rejection criterion, as well as the experimental procedures and planned analyses described below, were preregistered through the Open Science Foundation.^16^

##### Materials.

**Figure 21.**
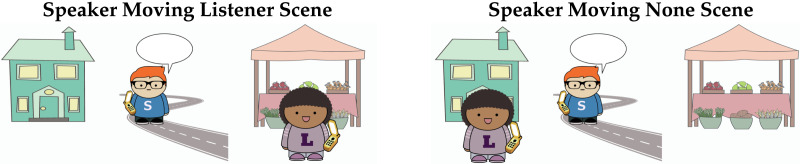
Speaker Moving scenes.

Two new scene conditions were added to the stimuli from Experiment 2a: a Speaker Moving Listener condition and a Speaker Moving None condition. These scenes show the speaker as the person in motion, and manipulate whether the listener is at the destination of motion (Figure 21).

##### Procedure.

Experiment 2b uses the same procedure as Experiment 2a, but extends the paradigm with two new scene conditions, for a total of 6 conditions: Both, Speaker, Listener, None, Speaker Moving Listener, and Speaker Moving None. Each participant saw 10 items in each condition, presented using a Latin Square design.

#### Data Coding.

The data coding procedure described for Experiment 2a was followed. The data was coded by an author and an annotator who was unaware of the purpose the experiment. Inter-annotator agreement scores ranged from 0.99 to 1.0 by condition.

#### Analysis.

A mixed-effects regression model was fit to the coded data as described for Experiment 2a, but with the two new Speaker Moving conditions added as fixed-effects, coded as follows: Speaker Moving Listener, 1 for the Speaker Moving Listener condition and 0 otherwise; and Speaker Moving None, 1 for the Speaker Moving None condition and 0 otherwise.

#### Results.

Table 9 shows the proportion of *come*, go, and manner-of-motion verbs used in descriptions of the motion event (2899 of the 3360 original responses). Raw responses are plotted in Figure 20.

**Table 9.** Experiment 2b proportion of motion responses by condition and type

**Mover**	**Scene**	***come***	***go***	***walk* etc**	***n* motion responses**
Thelma	Both	34%	17%	49%	468
	Speaker	32%	25%	43%	450
	Listener	6%	40%	54%	489
	None	1%	54%	45%	522

Speaker	Listener	14%	22%	64%	466
	None	0.2%	53%	47%	504

Overall, the results are similar to Experiment 2a. Though the proportion of *come* responses was higher in the Both condition than the Speaker condition, the difference was not significant in the mixed-effects model (Table 10). Thus, as in Experiment 2a, this experiment failed to find evidence of a Convergent Perspective Boost.

**Table 10.** Experiment 2b *come* response mixed effects logistic regression analysis

**Fixed effects (*n* = 3360)**	$\hat{\beta }$	***z***	***p***
(Intercept)	−1.62(±0.3)	−6.0	< **0.0001**
Both	0.12(±0.18)	0.7	0.50
Listener	−3.37(±0.4)	−7.7	< **0.0001**
None	−7.4(±1.3)	−5.6	< **0.0001**

Speaker Moving Listener	−1.3(±0.2)	−6.0	< **0.0001**
Speaker Moving None	−7.7(±2.1)	−3.7	**0.0002**

**Figure 22.**
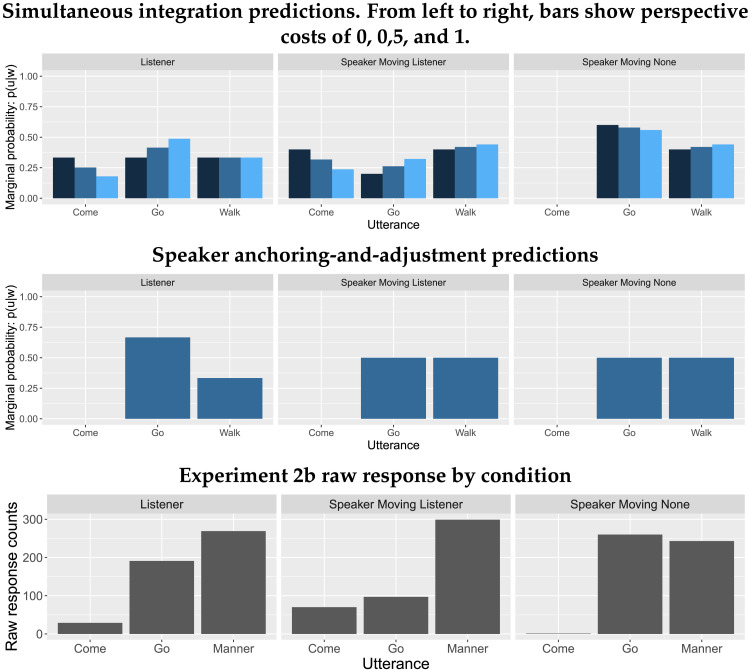
Speaker Moving production predictions and results.

Figure 22 shows the raw responses in the new Speaker Moving conditions, along with the predictions of the speaker anchoring-and-adjustment and simultaneous integration models. Participants produced *come* more frequently in the Speaker Moving Listener condition than in the Listener condition. The mixed-effects model finds a reliable difference between these two conditions (Table 11). This supports the existence of a Ambiguity Elimination Advantage, as predicted by the simultaneous integration model.

**Table 11.** Pairwise comparisons between Scene conditions in mixed effects logistic regression analysis

**Linear hypothesis**	$\hat{\beta }$	***z***	***p***
Both-Speaker==0	0.12(±0.2)	0.7	1.0
Listener-Speaker==0	−3.37(±0.4)	−7.7	< **0.001**
None-Speaker==0	−7.42(±1.3)	−5.6	< **0.001**
Speaker Moving Listener-Speaker==0	−1.26(±0.2)	−6.0	< **0.001**
Speaker Moving None-Speaker==0	−7.7(±2.1)	−3.7	**0.002**
Listener-Both==0	−3.49(±0.4)	−7.9	< **0.001**
None-Both==0	−7.5(±1.3)	−5.7	< **0.001**
Speaker Moving Listener-Both==0	−1.38(±0.2)	−6.1	< **0.001**
Speaker Moving None-Both==0	−7.8(±2.1)	−3.6	**0.002**
None-Listener==0	−4.04(±1.4)	−2.9	**0.028**
Speaker Moving Listener-Listener==0	2.11(±0.5)	4.6	< **0.001**
Speaker Moving None-Listener==0	−4.32(±2.1)	−2.0	0.25
Speaker Moving Listener-None==0	6.16(±1.3)	4.6	**0.001**
Speaker Moving None-None==0	−0.27(±2.5)	−0.1	1.0
Speaker Moving None-Speaker Moving Listener==0	−6.43(±2.1)	−3.1	**0.016**

#### Post-hoc Spatial Controls Analysis.

A post-hoc analysis of the spatial control items was performed as for Experiment 2a. Details can be found in Appendix A. For the Between and Close conditions, 88% of the responses were true according to the speaker’s perspective and not the visual perspective of the participant. 0.2% were true according the visual perspective of the participant and not that of the speaker character. We take this as evidence that participants were, on the whole, able to adopt the spatial perspective of the speaker character throughout the experiment.

### Combined Discussion

The production results suggest that speakers rely strongly on their own perspectives. No Convergent Perspective Boost was observed: the rate of *come* production in the Both condition was indistinguishable from the Speaker condition. Moreover, the low frequency of *come* in the Listener condition suggests that speakers strongly prefer their own perspectives to the listener’s.

The strength of the observed egocentric bias is consistent with the speaker anchoring-and-adjustment model of perspective selection, though, as noted above, it is also consistent with the simultaneous integration model under certain assumptions.

The results from the new conditions in Experiment 2b are easier to interpret: they support the simultaneous integration model. Speakers used *come* more often in the Speaker Moving Listener condition than in the Listener condition. This matches the simultaneous integration model’s prediction of an Ambiguity Elimination Advantage: *come* has higher utility when there are fewer potential perspective-holders for it. This finding is difficult to explain in the speaker anchoring-and-adjustment model, which expects speakers to maintain their own perspectives and use *go* or *walk*.

How can we reconcile these findings? We might imagine a rule-based system that augments the speaker anchoring-and-adjustment model with a preference to use *come* when possible. However, this variant of the speaker anchoring-and-adjustment model cannot explain the observed Ambiguity Elimination Advantage, since both the Listener scene and the Speaker Moving Listener scene can be felicitously described with *come*.

Thus, the production results provide conflicting evidence about grammatical perspective-taking in production. On the one hand, the signature prediction of the simultaneous integration model, the Convergent Perspective Boost, was not observed. On the other hand, the observed Ambiguity Elimination Advantage, which falls out naturally from the simultaneous integration model, is difficult to explain in the speaker anchoring-and-adjustment model.

While our work was under review, our production findings were replicated and extended by Watson et al. (2021). They report results from a web-based experiment with a larger sample size (*n* = 84) that measured motion verb production in the same conditions we explore in Experiment 2a (Both, Speaker, Listener, None). Unlike our production data, their data supports the existence of a Convergent Perspective Boost: their participants used *come* in the Both condition 20% of the time, compared to 13% in the Speaker condition. For other conditions, their data shows similar trends to ours.

The results from Watson et al. (2021) lend evidence in support of a simultaneous integration model of grammatical perspective-taking in production. However, they also raise the question of why a Convergent Perspective Boost was observed in their experiment, but not in ours. The difference in sample size is unlikely to account for it, since a meta-analysis combining the data from Experiments 2a and 2b (*n* = 96) does not change the patterns we report above. However, there may be other experimental design differences at play. The Watson et al. (2021) paradigm use maps that display the character’s motion path, rather than our two-location images. They also use automated text analysis tools rather than manual annotation to code their data.

One shared limitation of our work and Watson et al. (2021) is the use of a web-based design. Unlike much of the work on reference perspective-taking, these production tasks ask participants to imagine themselves as conversation participants, rather than interacting with an actual partner. Although our analysis of the spatial control items suggests that participants were able to adopt the perspective of their assigned character in the studies despite the web-based paradigm, it might make it harder for participants to access their (imagined) partner’s perspective.^17^ In an in-person experimental paradigm, we might therefore expect higher rates of perspective shift than found in our web-based experiments and those of Watson et al. (2021). This is an important avenue for future work.

## GENERAL DISCUSSION

In Modeling Grammatical Perspective-Taking section, we outlined two models of grammatical perspective-taking: the speaker anchoring-and-adjustment model, which posits a serial process with a strong bias towards speaker perspectives, and the simultaneous integration model, which posits that conversation participants consider multiple perspectives at once.

The comprehension experiments tested a key difference in the model predictions. The simultaneous integration model predicts a Convergent Perspective Boost: given a sentence with a perspectival expression, the listener should assign higher probability to scenes in which the sentence can be felicitously interpreted according to multiple perspectives. The speaker anchoring-and-adjustment model, on the other hand, predicts a Simple Speaker Advantage: all scenes where the utterance is compatible with the speaker’s perspective should be equally likely.

Taken together, the three comprehension experiments provided evidence of a Convergent Perspective Boost: comprehenders were faster to accept scenes where the speaker and listener were both located at the destination when interpreting an utterance with the perspectival motion verb *come*. This supports a simultaneous integration view of grammatical perspective-taking in comprehension.

The production experiments tested two predictions of the simultaneous integration model: the Convergent Perspective Boost and the Ambiguity Elimination Advantage. According to the Ambiguity Elimination Advantage, speakers should use perspectival expressions more often when ambiguity over the perspective-holder is reduced. This follows from the way that the simultaneous integration model incorporates reasoning over the listener’s interpretive process. By contrast, the speaker anchoring-and-adjustment model expects the listener to always select the same perspective as the speaker (the speaker’s perspective), and does not predict an advantage for eliminating perspective ambiguity.

The production results were mixed. No evidence of a Convergent Perspective Boost was observed, contrary to the simultaneous integration model’s prediction. However, an Ambiguity Elimination Advantage effect was observed in Experiment 2b, which cannot be easily explained by the speaker anchoring-and-adjustment model. In addition, a subsequent production experiment by Watson et al. (2021) using a similar web-based paradigm as ours did find evidence of a Convergent Perspective Boost in production.

A key finding from the experiments presented above is an apparent asymmetry between grammatical perspective-taking in production and comprehension. Although the response measures in the two experiments are not directly comparable, since production participants were free to generate a wider range of responses than comprehension participants saw, there appears to be an asymmetry between the comprehension and production results: the comprehension results supported the existence of a Convergent Perspective Boost, while the production results did not.

We see two directions to explore with the aim of reconciling these findings. The first is to adopt the simultaneous integration model for comprehension, and the speaker anchoring-and-adjustment model for production. This is compatible with the two-stage system proposed by Harris (2012): perhaps both systems are in principle available for speakers and listeners, but listeners are more motivated to use the costlier reasoning system than speakers. The second is to reconcile the production results into a simultaneous integration model.

We argue in favor of the second approach, with some reservations, for two reasons. First, a speaker anchoring-and-adjustment model cannot fully account of the patterns we observe in production. If speakers are relying on a simpler heuristic-based grammatical perspective-taking system, then we should not observe an Ambiguity Elimination Advantage in production, since this arises only from reasoning over multiple available perspectives. Although Experiment 2b did not reveal evidence of a Convergent Perspective Boost, speakers used *come* significantly more often in the Speaker Moving Listener condition than the Listener condition, which cannot be easily explained by a model that uses a simple speaker-default heuristic.

Second, as noted in Production Experiments section, with a high perspective cost, the predicted Convergent Perspective Boost becomes very small, making it hard to distinguish between the simultaneous integration and speaker anchoring-and-adjustment models. Moreover, subsequent work has found evidence of a Convergent Perspective Boost in production. Although it is unclear why Watson et al. (2021) and our findings diverge, they do find a Convergent Perspective Boost in a fairly similar experimental paradigm, which suggests that speakers are reasoning over multiple perspectives simultaneously in some contexts. This is, again, compatible with a two-stage system like the one sketched in Harris (2012), since it could be that speakers are more motivated to use the costlier reasoning system in Watson et al. (2021)’s experimental paradigm than they are in ours. However, if this is so, it is important to understand why our contexts differ.

The question of what is driving the key difference between our results and Watson et al. (2021)’s findings is a serious one. The fact that relatively minor differences in our paradigms (the use of maps versus scenes, the inclusion of an Uncertainty manipulation, differences in data coding) have led to such different findings is troubling, and highlights the need for more work on grammatical perspective-taking in a wider range of contexts and with different experimental paradigms. Replicating our work in a dyadic, in-person context would be a particularly important next step towards reconciling our findings.

Our finding of an Ambiguity Elimination Advantage effect in Experiment 2b in tandem with the fact that Watson et al. (2021)’s subsequent work finds evidence of Convergent Perspective Boost in production leads us to adopt the simultaneous integration model for both comprehension and production. However, the observed asymmetry in our data remains to some extent: our production participants use their own perspectives more frequently than our comprehension participants appear to expect them to. What should we make of this?

One possibility is that it is an artifact of whatever experimental design choices lead our production findings to differ from those in Watson et al. (2021). Since Watson et al. (2021) do not include a comprehension task, we cannot determine whether the differences between our experimental stimuli would have also had an impact on listener behavior. This makes it hard to know whether the asymmetry we observe would remain even if we observed a Convergent Perspective Boost in production as in Watson et al. (2021). Do speakers demonstrate a heavier bias towards their own perspectives than listeners expect them to?

If there is, in fact, an asymmetry in how speaker-biased speakers and listeners are, it could be accounted for under a symmetrical model like the PRSA simultaneous integration model, in tandem with an asymmetric cognitive bias. We find that speakers are strongly biased towards their own perspectives, while listeners display a weaker bias towards the speaker’s perspective. Perhaps this results from egocentric bias in both directions: speakers are strongly biased towards their perspectives, while listeners have a weaker self-perspective bias. In this case, the underlying model would need to include a moderate speaker bias that is strengthened in production by the speaker’s egocentric bias and weakened in comprehension by the listener’s egocentric bias.

The asymmetry between speaker and listener behavior that we observe is paralleled in other domains. Ryskin et al. (2020) find that in reference perspective-taking, speakers consider the Common Ground perspective less than listeners do. Kehler and Rohde (2013, 2019) similarly find an asymmetry in pronoun production and comprehension, where listener expectations about which individuals will be pronominalized do not match speaker behavior.

These asymmetries are puzzling on a strongly Bayesian view of comprehension and production, where speakers and listeners base their behavior on mental models of each others’ behavior that they are constantly updating. In this view, we would expect speaker and listener estimates of each others’ egocentric biases to converge, and any discrepancies in their expectations about each others’ behavior to gradually disappear. This is true regardless of how the egocentric bias is interpreted: if it applies to listeners as well as speakers, over time, speakers should adapt their production model accordingly, and vice versus.

Kehler and Rohde (2019) lay out one promising path towards reconciling the Bayesian view with the observed production/comprehension asymmetries across domains. They propose for pronominalization that listeners have access to additional evidence in pronoun resolution beyond what speakers use in pronoun production. In their model, pronoun production is primarily driven by grammatical or structural information such as topicality and subjecthood (in line with centering views of pronominalization; Grosz et al., 1995). Pronoun comprehension, meanwhile, takes into account additional semantic and pragmatic factors like verb type and discourse coherence (in line with coherence views of pronominalization; Hobbs, 1979).

If subsequent work confirms the existence of an asymmetry between grammatical perspective-taking in production and comprehension, this would be one avenue to explore. Many of the factors proposed to influence pronominalization and pronoun resolution are also known to affect perspective prominence (Abrusán, 2021; Bimpikou, 2020; Hinterwimmer, 2019; Kaiser, 2018; Kaiser & Lee, 2017a, 2017b).^18^ Thus, Kehler and Rohde (2019)’s findings for pronominalization might generalize to grammatical perspective-taking and explain the production/comprehension asymmetry that we observed: speakers do not include the same kinds of evidence in their perspective selection process as listeners consider in perspective inference.

In the remainder of this section, we discuss the comprehension and production findings together in the broader context of perspective-taking.

### Egocentricity in Grammatical Perspective-Taking

One finding that emerges from the combined comprehension and production results is a strong bias towards the speaker’s perspective. In both comprehension and production, the listener’s perspective is much less accessible than the speaker’s. In comprehension, the acceptance rate of the Listener scene was around 60–65%, suggesting that about a third of the time, participants did not consider that the speaker might have adopted the listener’s perspective. In production, participants produced *come* in the Listener scene around 5% of the time.

Both models that we consider can account for this speaker bias, though in different ways. It falls out naturally from the speaker anchoring-and-adjustment model, since the speaker’s perspective is always used if compatible. In the simultaneous integration model, these results are accounted for by setting a high penalty for non-speaker perspectives in the perspective cost function.

The source of the speaker bias remains an open question. Some treatments of grammatically perspectival expressions have stipulated a speaker-orientation in the semantics of the expressions themselves, and accounted for other uses by some grammatical mechanism (such as context-shifting) (Potts, 2005; Sudo, 2018). However, this is problematic for come, which allows listener-oriented interpretations in a wide variety of grammatical environments. Alternatively, speaker bias might arise as a result of a general cognitive bias towards self perspectives. This is plausible given that such egocentric bias has been observed in perspective-taking in reference contexts where there are no lexically perspectival expressions.

Our results provide only partial support for a more general bias. It is unclear why this bias would apply more strongly in production than in comprehension, since our comprehension participants were also biased towards the speaker’s perspective, rather than towards their own. Past work on egocentric bias in the reference perspective-taking domain finds that listeners are accordingly biased to their own perspectives (Epley, Keysar, et al., 2004). Yet we observe a strong preference for speaker perspectives in comprehension as well, where a self bias should lead to a preference for the listener’s perspective, rather than the speaker’s. Our comprehension results are more compatible with a view in which egocentricity is a speaker bias that listeners are aware of and incorporate into their mental model of the speaker.

### Perspective-Taking in Conversation

Our findings align well with the emerging picture of perspective-taking in reference. However, we observe some key differences between the two perspective-taking domains. As in work on perspective-taking when producing referring expressions, we find evidence of a bias towards the speaker’s perspective. However, our production results suggest a much stronger bias than has been observed in recent work on reference perspective-taking. In the production data presented by Hawkins et al. (2021), speakers almost always take into account the listener’s perspective. Ryskin et al. (2020)’s re-analysis of the data from Mozuraitis et al. (2018) suggests a moderate egocentric bias, more in line with what we observe.^19^ However, their re-analysis of the comprehension data from Heller et al. (2016) suggests an egocentric bias on the part of the listener towards their own perspective, which we do not observe in our comprehension results.

It is important to keep in mind the differences between the two domains of perspective-taking, and the experimental paradigms that have been employed to investigate them. Reference perspective-taking is not an all-or-nothing proposition: the speaker and the listener’s shared information can be represented as a Common Ground perspective. The egocentric biases quantified in Ryskin et al. (2020) represent failures to take into account the information asymmetry between the conversation partners. In grammatical perspective-taking, there is no such Common Ground perspective: there is a grammatical element that must be resolved to a single perspective-holder in the discourse context.

The motivation to shift perspective also varies with the experimental paradigm. In the reference perspective-taking paradigm, the speaker knows that failure to consider the listener’s perspective (information state) is likely to lead to a miscommunication. Failing to take the listener’s perspective in our production experiment, on the other hand, is less likely to cause confusion, since the speaker has other strategies of clarification available: they can use a manner-of-motion verb to avoid perspectival reasoning altogether, or specify extra information about the scene. Moreover, our experiment did not incorporate an explicit failure signal (like selection of the wrong object).

Our web-base paradigm likely provided less motivation to participants to consider their conversation partner’s perspective than an in-person experiment with a physically present participant would; this remains an important area for future work.

Despite the key differences discussed above, there are two shared patterns of interest in our findings for grammatical perspective-taking and recent findings for reference perspective-taking: the asymmetry between comprehension and production discussed above, and interspeaker variability in perspective access.

### Interspeaker Variability

In their work on perspective-taking in reference, Ryskin et al. (2020) find a high degree of variability in the degree of egocentricity for different production participants. A sizeable group of participants (∼25) almost never took into account their listeners’ perspectives. A smaller group (∼7) almost always took their listeners’ perspectives. The egocentric biases of the remaining participants (∼100) were spread between these two extremes.

We also observe a fair amount of variability in the accessibility of the speaker perspective in both production and comprehension. Although a large group of participants in the comprehension experiments accept all listener scenes with *come* (∼127), a small group never accept them (∼21), and the rest of the participants accept them with some frequency (∼125). The production results are generally more variable given the less constrained nature of the task.

This finding aligns with other experimental work on grammatical perspective, which suggests that the rate of access to non-speaker perspectives is highly variable across participants (Anderson, 2019; Bimpikou, 2020; Duff, 2018).

### Threats to Validity

The differences between our findings and those in the reference perspective-taking domain may also be due to differences in our experimental paradigms. An advantage of Mozuraitis et al. (2018)’s experimental design is that it used in-person speaker-listener participant pairs. Our web-based paradigm might have made it more difficult for participants to adopt the relevant perspectives.

Our task asks participants to imagine themselves as one of the participants in a conversation. One threat to the external validity of our experiment comes from this aspect of the task. In a real conversation, of course, each conversation partner does not need to imagine themselves in a particular setting: they are located in that setting. Our task is therefore more akin to what happens in complicated uses of grammatically perspectival expressions, such as reported speech and narrative processing, than in simple dyadic conversations.

We measure the extent to which participants are able to adopt the perspectives of the characters they were instructed to imagine themselves as using our spatial control task. In the comprehension experiments, participants were excluded from the study if they did not demonstrate the ability to adopt the spatial perspective of the listener character. Due to the open-ended nature of the production experiments, the spatial control items were not used as an exclusion mechanism. However, our post-hoc data analysis reveals that participants rarely used their own visual perspective on the scene, and described the scene according to the speaker’s perspective in most cases.

Although we establish that the participants are capable of adopting the character’s perspectives, using a dyadic in-person paradigm might change their behavior in other ways. For instance, interacting with a human interlocutor could increase participants’ motivation to communicate successfully. This might lead them to adopt their interlocutor’s perspective more often. In the reference perspective-taking domain, Yoon et al. (2012) find that speakers take into account the listener’s perspective more often when the speaker is making a request rather than a statement, and Brown-Schmidt (2009) demonstrates that interactive tasks stimulate more consideration of a partner’s perspective than non-interactive tasks. Replicating our findings in an interactive dyadic paradigm is therefore a critical direction for future work.

## CONCLUSION

Although grammatically perspectival expressions are cross-linguistically common, relatively little is known about how they are processed. In this paper, we have contributed novel evidence about the production and comprehension of one class of grammatically perspectival expressions: perspectival motion verbs like *come* and *go*. We have explored two kinds of models that have been much discussed in work on reference perspective-taking: a serial, speaker-biased model that we call the speaker anchoring-and-adjustment model, and a simultaneous integration model, where conversation participants consider multiple perspectives at once.

Our comprehension results support a simultaneous integration view of grammatical perspective-taking: listeners appear to consider multiple perspectives at once when interpreting grammatically perspectival expressions. However, our production results are more mixed: we find no evidence of the Convergent Perspective Boost observed in comprehension, but we do find evidence of a Ambiguity Elimination Advantage, which is difficult to account for in a serial model of perspective selection like the speaker anchoring-and-adjustment model.

Taking into consideration our own data and the subsequent experimental work by Watson et al. (2021), we argue for a simultaneous integration model in both comprehension and production, providing experimental evidence from grammatical perspective-taking to support the proposal that conversation participants reason jointly over multiple perspectives (Anderson & Dillon, 2019; Harris, 2012), and paralleling findings for perspective-taking in reference (Hawkins et al., 2021; Heller et al., 2016; Mozuraitis et al., 2018; Ryskin et al., 2020). However, the explanation for the key difference in our production data and those of Watson et al. (2021) remains unclear, motivating further work on grammatical perspective-taking in a wider variety of contexts, and with different experimental paradigms.

Taken as a whole, our experiments reveal patterns in grammatical perspective-taking that echo those in other domains of perspective-taking. We find that both speakers and listeners have a preference for speaker-oriented perspectival expressions, but that speakers are more strongly biased towards their own perspectives. Despite the differences between domains, the existence of a strong speaker bias and the evidence in support of simultaneous perspective integration parallel findings in related domains like reference perspective-taking and pronoun resolution, suggesting that they may spring from more general properties of production and comprehension.

## ACKNOWLEDGMENTS

The authors thank Rajesh Bhatt, Daniel Altshuler, Mohit Iyyer, and the anonymous reviewers at *Open Mind* for their thoughtful feedback on the experimental results and computational modeling. Thanks are also due to Judith Degen, Lyn Frazier, Richard Futrell, Jesse Harris, and Daphna Heller for fruitful discussion. This work has benefited from discussion at the 2019 annual meeting of the Society for Computation in Linguistics and Rational Approaches in Language Science 2019.

## Notes

^1^ The set of licit perspective-holders varies by language; see Gathercole (1987), Nakazawa (2007), and Barlew (2017) for further discussion of what is known about cross-linguistic variation.^2^ In the perspective-anaphoric treatment we adopted in Modeling Grammatical Perspective-Taking section. Alternative analyses of perspectival motion verbs also involve a perspectival variable, but at different levels of representation: indexical approaches posit a perspectival field in the context parameter (Lasersohn, 2005; Oshima, 2006a), and logophoric binding accounts posit a logophoric pronoun that binds the perspectival variable (Charnavel, 2020).^3^ Harris’s heuristic system also incorporates a preference for the last-used perspective; since the simulations and experiments that we present do not involve multiple perspectival expressions, this is less relevant for the present investigation.^4^ See Anderson (2021) for an argument that the production domain does not provide sufficient evidence to motivate the kind of switch Harris (2012) posits for comprehension.^5^ https://osf.io/3bsnz/?view_only=1091b1607b864161860f84dcafe3e425.^6^ https://osf.io/3bsnz/?view_only=1091b1607b864161860f84dcafe3e425.^7^ https://aspredicted.org/blind.php?x=xa47j8.^8^ The manner-of-motion descriptions used were: *walking*, *driving*, *on the way*, *en route*, *skateboarding*, and *headed to*.^9^ In Experiment 1a, the average accuracy on the Close and Between conditions for participants not excluded from the study was 97%, compared to 91% for the left/right condition.^10^ https://osf.io/3bsnz/?view_only=1091b1607b864161860f84dcafe3e425.^11^ We assume here that the speaker uses the same perspective selection process in both cases; it could be that the speaker’s production process is different when they are the mover, however.^12^ https://osf.io/3bsnz/?view_only=1091b1607b864161860f84dcafe3e425.^13^ This is important because many perspectival expressions are known to undergo *interrogative flip*: in questions, the default perspective-holder shifts from the speaker to the listener (Faller, 2002).^14^ Our data coders used the following rule of thumb to determine light verb uses of *go*: “Substitute in *walk* for *go* and see whether the resulting sentence makes sense. For instance, *Thelma is going to come to the store* fails this test because *Thelma is walking to come to the store* is odd, while *Thelma is going to get coffee* passes because *Thelma is walking to get coffee* makes sense.” This errs on the side of including ambiguous future auxiliary uses of *go* as actual motion events, since *Thelma is going to get coffee* could describe either a current or future event (*Thelma is going to get coffee with Margaret next week*).^15^ We preregistered a model that used treatment coding with the Speaker condition as the baseline. We switched to this model because the low rate of *come* responses in the Listener condition motivated a model that could compare the Listener and None conditions. The reliable effects are the same under both.^16^ https://osf.io/3bsnz/?view_only=1091b1607b864161860f84dcafe3e425.^17^ Hawkins et al. (2021)’s web-based reference perspective-taking work finds lower rates of egocentricity than Heller et al. (2016)’s lab-based work, but perhaps this counter-intuitive discrepancy is explained by other task factors.^18^ In fact, in the perspective-anaphoric analysis of *come* that we use, following Barlew (2017), perspective inference is a kind of pronoun resolution.^19^ Though, given the differences in the experimental paradigms, it is difficult to compare their estimates directly with ours.^20^ Data coding guidelines and annotated data can be found in the Open Science Foundation repository for this project: https://osf.io/3bsnz/?view_only=1091b1607b864161860f84dcafe3e425.

## APPENDIX A: POST-HOC ANALYSIS OF SPATIAL CONTROLS

To explore whether the ability of participants to take on the speaker’s perspective poses a threat to the external validity of our task, a post-hoc analysis of the spatial control items was performed. Responses to these items were coded 1 if they were true according to the speaker’s perspective and not the scene viewer’s perspective; −1 if they were true according to the scene viewer’s perspective and not the speaker’s, and 0 if they did not provide evidence in either direction.

These data coding guidelines were not preregistered, and the data coding in this post-hoc analysis was performed by only one annotator, unlike the main items, which were coded by an author and a second annotator who did not know the purpose of the experiment.^20^

### Experiment 2a

For the Between and Close conditions, 89% of the responses were true according to the speaker’s perspective and not the visual perspective of the participant. 0.5% were true according the visual perspective of the participant and not that of the speaker character. For the Right/Left condition, 72% of the responses were true according to the speaker’s perspective and not the visual perspective of the participant. 6% were true according the visual perspective of the participant and not that of the speaker character. Although a number of the responses fall into the ambiguous category, this shows that in the majority of trials, participants were adopting the speaker character’s perspective rather than relying on their own visual perspective.

### Experiment 2b

For the Between and Close conditions, 88% of the responses were true according to the speaker’s perspective and not the visual perspective of the participant. 0.2% were true according the visual perspective of the participant and not that of the speaker character. For the Right/Left condition, 64% of the responses were true according to the speaker’s perspective and not the visual perspective of the participant. 14% were true according the visual perspective of the participant and not that of the speaker character. Although the participants in Experiment 2b struggled more with the Right/Left condition than those in Experiment 2a, we take this as evidence that participants were, on the whole, able to adopt the spatial perspective of the speaker character throughout the experiment.
